# Imaging and multi-omics datasets converge to define different neural progenitor origins for ATRT-SHH subgroups

**DOI:** 10.1038/s41467-023-42371-7

**Published:** 2023-10-20

**Authors:** María-Jesús Lobón-Iglesias, Mamy Andrianteranagna, Zhi-Yan Han, Céline Chauvin, Julien Masliah-Planchon, Valeria Manriquez, Arnault Tauziede-Espariat, Sandrina Turczynski, Rachida Bouarich-Bourimi, Magali Frah, Christelle Dufour, Thomas Blauwblomme, Liesbeth Cardoen, Gaelle Pierron, Laetitia Maillot, Delphine Guillemot, Stéphanie Reynaud, Christine Bourneix, Célio Pouponnot, Didier Surdez, Mylene Bohec, Sylvain Baulande, Olivier Delattre, Eliane Piaggio, Olivier Ayrault, Joshua J. Waterfall, Nicolas Servant, Kevin Beccaria, Volodia Dangouloff-Ros, Franck Bourdeaut

**Affiliations:** 1 INSERM U830, Laboratory of Translational Research In Pediatric Oncology, PSL Research University, SIREDO Oncology center, Institut Curie Research Center, Paris, France; 2 INSERM U900, Bioinformatics, Biostatistics, Epidemiology and Computational Systems Unit, Institut Curie, Mines Paris Tech, PSL Research University, Institut Curie Research Center, Paris, France; 3 Somatic Genetic Unit, Department of Pathology and Diagnostic and Theranostic Medecine, Institut Curie Hospital, Paris, France; 4 INSERM U932, Immunity and Cancer, PSL Research University, Institut Curie Research Center, Paris, France; 5 Department of Neuropathology, GHU Paris-Psychiatry and Neurosciences, Sainte-Anne Hospital, Paris, France; 6 Paris Psychiatry and Neurosciences Institute (IPNP), UMR S1266, INSERM, IMA-BRAIN, Paris, France; 7 Department of Children and Adolescents Oncology, Gustave Roussy, Paris Saclay University, Villejuif, France; 8 Department of Pediatric Neurosurgery-AP-HP, Necker Sick Kids Hospital, Université de Paris, Paris, France; 9 Imaging Department, Institut Curie Hospital, Paris, France; 10 CNRS UMR 3347, INSERM U1021, Institut Curie, PSL Research University, Université Paris-Saclay, Orsay, France; 11 INSERM U830, Diversity and Plasticity of Childhood Tumors Lab, PSL Research University, SIREDO Oncology Center, Institut Curie Research Center, Paris, France; 12 Balgrist University Hospital, Faculty of Medicine, University of Zurich (UZH), Zurich, Switzerland; 13 Institut Curie, PSL University, Single Cell Initiative, ICGex Next-Generation Sequencing Platform, PSL University, 75005 Paris, France; 14 INSERM U830, Integrative Functional Genomics of Cancer Lab, PSL Research University, Institut Curie Research Center, Paris, France; 15 Department of Translational Research, PSL Research University, Institut Curie Research Center, Paris, France; 16 Pediatric Radiology Department, AP-HP, Necker Sick Kids Hospital and Paris Cite Universiy INSERM 1299 and UMR 1163, Institut Imagine, Paris, France; 17 Department of Pediatric Oncology, SIREDO Oncology Center, Institut Curie Hospital, Paris, and Université de Paris, Paris, France

**Keywords:** Embryonal neoplasms, CNS cancer

## Abstract

Atypical teratoid rhabdoid tumors (ATRT) are divided into MYC, TYR and SHH subgroups, suggesting diverse lineages of origin. Here, we investigate the imaging of human ATRT at diagnosis and the precise anatomic origin of brain tumors in the Rosa26-Cre^ERT2^::Smarcb1^flox/flox^ model. This cross-species analysis points to an extra-cerebral origin for MYC tumors. Additionally, we clearly distinguish SHH ATRT emerging from the cerebellar anterior lobe (CAL) from those emerging from the basal ganglia (BG) and intra-ventricular (IV) regions. Molecular characteristics point to the midbrain-hindbrain boundary as the origin of CAL SHH ATRT, and to the ganglionic eminence as the origin of BG/IV SHH ATRT. Single-cell RNA sequencing on SHH ATRT supports these hypotheses. Trajectory analyses suggest that *SMARCB1* loss induces a de-differentiation process mediated by repressors of the neuronal program such as *REST*, ID and the NOTCH pathway.

## Abstract (web summary)

Atypical teratoid rhabdoid tumors (ATRT) are divided into three molecular subgroups, which could have different lineages of origin. Here, the authors use tumour imaging, multi-omics and genetically engineered mouse models to determine the anatomical region and cell lineage of origin of ATRT subtypes.

## Introduction

Atypical teratoid rhabdoid tumors (ATRT) are rare and aggressive malignancies of the central nervous system (CNS) affecting infants and young children. They are characterized by a biallelic inactivation of *SMARCB1* tumor suppressor gene in an otherwise very stable genome^1^. Based on both methylation and expression profiling, recent studies have highlighted the molecular diversity of these tumors^2,3^, which are now divided into at least three subgroups, i.e., the so-called MYC-, TYR-, and SHH-ATRTs^4^. This molecular diversity suggests multiple lineages of origin for each sub-type, though none of these origins is definitively identified at the present time. In this respect, the analysis of expression profiling has been weakly informative, giving at most some insights on the lineage (neurogenic or melanogenic features for the SHH and TYR subtypes respectively) or on some recurrently expressed developmental genes and pathways (SHH and NOTCH pathway for the SHH subtype; HOX clusters for the MYC subtype). Methylation profiling analyses including ATRT as well as extra-cranial rhabdoid tumors (ECRT) have revealed that MYC ATRT and ECRT tend to cluster together, apart from TYR and SHH ATRT, suggesting that MYC ATRT may share some commonalities with tumors that emerge outside of the brain^5^.

These results are consistent with single-cell RNA sequencing (scRNAseq) results failing to find any convincing correlation between tumor cells from MYC ATRT and any known cell types from normal brain development^6^. These data were consistent with our own findings in an inducible mouse model, where gene expression profiling of mouse Myc ATRT suggested a neural-crest-derived lineage^7^. Likewise, a genotyping approach, comparing tumor cells with adjacent normal tissues, suggested that ECRT derive from ancestors shared with Schwann cells, thus pointing to a neural-crest-derived origin^8^. These results were also partly supported by the development of typical ATRT-like tumors in P0-Cre::Smarcb1^flox/flox^ mouse, again pointing to neural-crest cells as putative cells of origin^9^. Finally, applying scRNAseq to murine tumors, Graf et al. have suggested that primordial germ cells could be the origin for at least a subset of murine Myc ATRT^10^. Altogether, while a neuronal origin is considered for SHH ATRT and a non-neuronal origin for MYC ATRT, the question of the lineage of origin of each ATRT subtype remains mostly speculative.

In the present study, we thoroughly investigated the imaging at diagnosis of a series of primary ATRT, aiming to describe the epicenter and thereby, the site of origin for each case. We next correlated these findings with the molecular profiles, including the new imaging results in the definition of the subgroups together with methylation and expression profiling. Finally, we performed a similar approach on tumors from the Rosa26-Cre^ERT2^::Smarcb1^flox/flox^ model, and added a single-cell RNAseq analysis on both human and mouse tumors to investigate the putative cells of origin for the various ATRT subtypes.

## Results

### Radiological description of ATRTs’ epicenter suggests clearly distinct origins for each molecular subtype

**Fig. 1:**
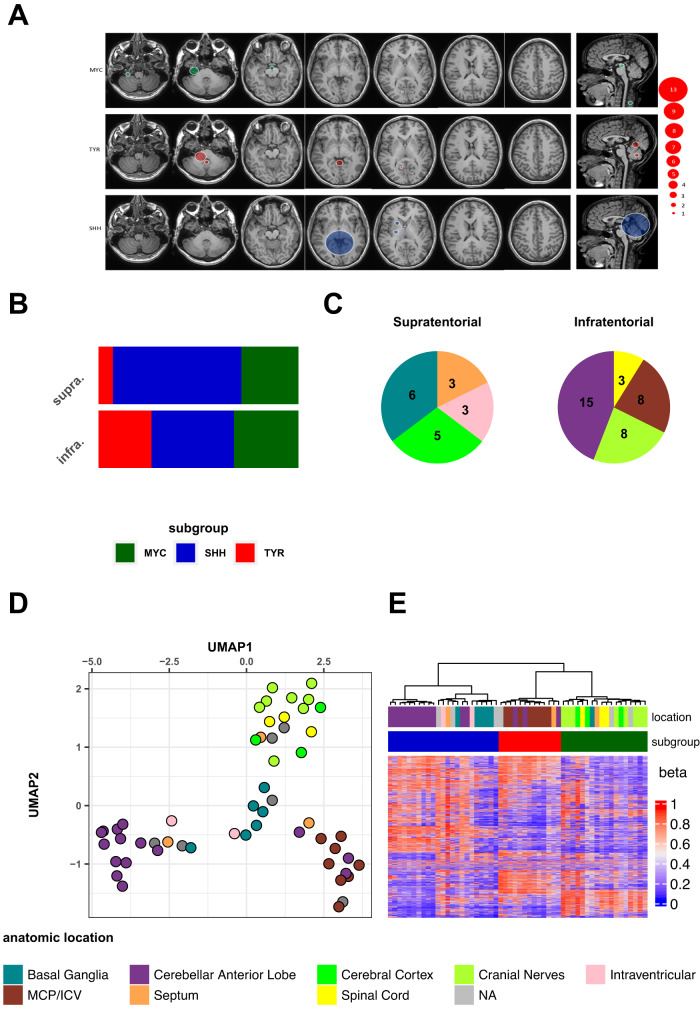
Radiological description of ATRTs’ epicenter suggests clearly distinct origins for each molecular subtype. **A** MRI showing the most frequent tumor locations according to molecular subgroups. The round size indicates the number of tumors. The last column represents the tumors located on the midline, which are not additional cases, except the spinal MYC tumors. The different colors correspond to the molecular subgroups based on DNA methylation data (DKFZ brain tumor classifier v11b4): MYC (green), TYR (red) and SHH (blue). **B** Bar plot showing the distribution of the different molecular groups assigned according to the DNA methylation profile at the supra- and infratentorial level. **C** Pie charts showing the distribution of the different ATRT anatomical locations at infra and supratentorial levels. NA: not available anatomical location. The color code is referred to at the bottom of Fig. 1. MCP/ICV: middle cerebellar peduncle and inferior cerebellar vermis. **D** UMAP analysis performed on human ATRT DNA methylation array data. **E** Unsupervised hierarchical clustering of ATRT samples based on DNA methylation data. Top annotation indicates ATRT anatomical location and molecular subgroup. Source data are provided as a Source Data file.

We first reviewed a series of 54 human brain ATRT (Supplementary Fig. 1A; Supplementary Data 1), aiming to categorize them following the classically used locations, i.e. infratentorial or supratentorial. Using the online DKFZ brain tumor DNA methylation-based classifier tool^11^ (http://www.molecularneuropathology.org/), we also assigned a molecular subgroup to this series and found that, while the infratentorial location is not per se suggestive of any tumor subgroup, the supratentorial location could suggest both MYC or SHH subtypes (Fig. 1A, B; Supplementary Data 1). For a higher-resolution analysis of anatomic location, we then categorized the tumors from their presumptive epicenter, aiming to give a more precise anatomic origin. This led us to define eight anatomical categories of ATRT: (i) cranial nerves, tumors from extra-axial structures such as interpeduncular cistern (IIIrd cranial nerve), internal acoustic canal (VII/VIIIth cranial nerves), cavernous sinus (IIIrd, IVth and Vth nerves) and jugular foramen, (IX/X/XIth nerves); (ii) cerebellar anterior lobe (CAL), tumors spreading from the quadrigeminal cistern to the anterior vermis; (iii) tumors from the middle cerebellar peduncles and inferior cerebellar vermis (MCP/ICV); (iv) peripheral tumors located in the cerebral cortex, pressing the normal parenchyma towards the ventricles; (v) intraventricular (IV) tumors, which were often large tumors in close relationship with the basal ganglia region; (vi) basal ganglia (BG) tumors, centered on the basal ganglia, pushing the brain parenchyma to the periphery; (vii) septal tumors, located in the interventricular septum; and finally, (viii) spinal cord tumors (Supplementary Fig. 1B). Overall, we ended up with four locations belonging to the infratentorial region and four locations belonging to the supratentorial region (Fig. 1C).

Combining these detailed anatomic locations with the DNA methylation subgroups, we found different localization patterns for each molecular subtype. Specifically, (i) almost all TYR ATRT emerged from the middle cerebellar peduncle and the inferior cerebellar vermis; (ii) the SHH subgroup was composed of tumors located in the CAL, the BG and the intraventricular region; and (iii) the MYC subgroup was mainly composed by tumors located in the cranial nerves, in the cerebral cortex and the spinal cord (Supplementary Fig. 1C). Altogether, these findings showed a correlation between anatomical location and molecular subgroups that suggested different lineages of origin. To further investigate this, we performed unsupervised analyses (UMAP and hierarchical clustering) on this DNA methylation dataset. We found that ATRT samples clustered according to their anatomical location (Figs. 1D, E). Furthermore, the SHH subgroup seemed to be composed of two distinct sub-clusters: one composed almost exclusively of tumors from CAL and the other mainly composed of BG and IV tumors (Fig. 1E).

### Integrative analysis identified four anatomical-molecular subgroups and splits SHH ATRT in two subgroups with distinct anatomical locations and transcriptional profiles

**Fig. 2:**
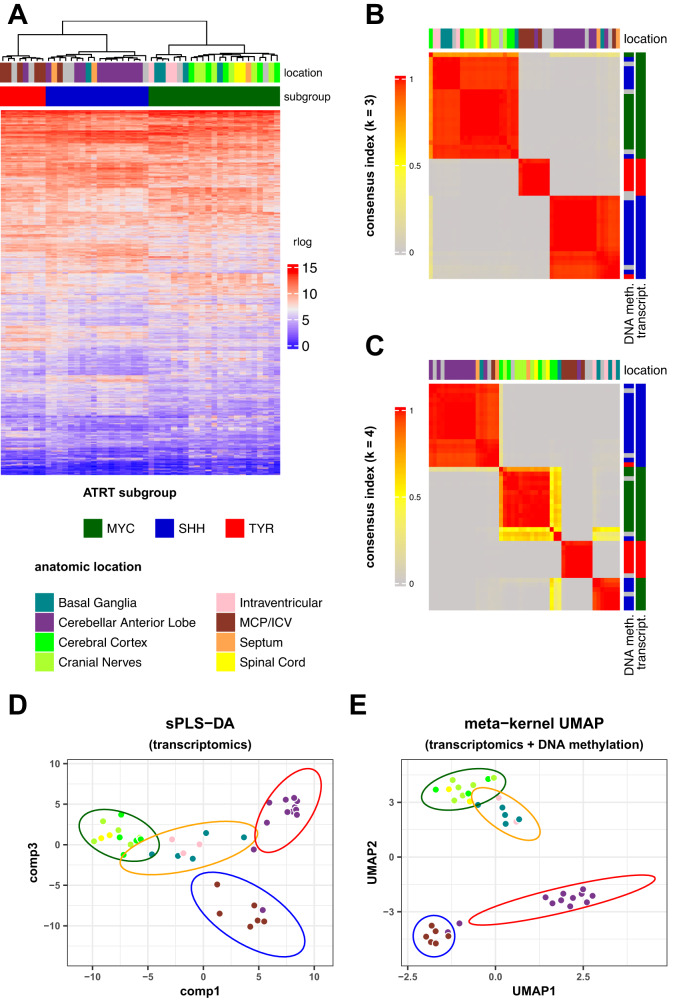
Integrative analysis identified four anatomical-molecular subgroups and splits SHH ATRT in two subgroups with distinct anatomical locations and transcriptional profiles. **A** Unsupervised hierarchical clustering of human ATRT samples based on RNAseq dataset. Anatomic location as well as the assigned RNAseq subgroup for each sample is indicated in the top annotations. **B**, **C** Consensus clustering of human ATRT samples based on RNAseq data with *k* = 3 (**B**) and *k* = 4 (**C**). **D** UMAP of human ATRT based on the integrated DNA methylation and transcriptomics datasets (kernel-based approach). Points indicate tumor samples, colors indicate anatomic location and the ellipses indicate tumor anatomical-molecular subgroups. **E** sPLS-DA individual plot using the two major components (comp, comp 1, and comp3). Points indicate tumor samples, colors indicate anatomic location and the ellipses indicate tumor anatomical-molecular subgroups. sPLS-DA was applied on RNAseq data. Source data are provided as a Source Data file.

Next, we sought to explore how the anatomically defined ATRT classification behaved at the transcriptomic level. We first performed an unsupervised analysis on the RNAseq data from 49 samples based on the 2000 most variable genes. Hierarchical clustering showed three molecular subgroups that corresponded to TYR, SHH, and MYC (Fig. 2A).

To achieve a more comprehensive subgrouping, we next performed a data integration approach using the three layers of information: DNA methylation, gene expression, and anatomical location. We then performed consensus clustering based on the transcriptomic dataset (Fig. 2B, C, Supplementary Fig. 2A). Assuming the three subtypes, we found some discrepancy between methylation and RNAseq subgroupings (Fig. 2B); however, the consensus clustering solved this discrepancy with *k* = 4 by separating BG/IV (SHH methylation signature but MYC gene expression profile) from CAL SHH ATRT (SHH by methylation and transcriptomic signatures) and MYC (MYC by both methylation and transcriptomic signatures) (Fig. 2C). Sparse Partial Least Square Determinant Analysis (sPLS-DA) applied to RNAseq also supported the four subgroups (Fig. 2D, Supplementary Fig. 2B) and allowed us to identify genes that were specific to each subgroup (Supplementary Data 2). Consistently, a kernel-based data integration method^12^, aiming to combine gene expression and DNA methylation datasets before unsupervised analysis, confirmed the existence of four molecular subgroups that fitted with our anatomical classification (Fig. 2E). These results established that the SHH ATRT, canonically defined by DNA methylation, could be divided in two distinct subgroups, with distinct gene expression profiles and specific anatomic locations.

### Genetically engineered mouse models reveal specific anatomical origins for murine Myc and Shh ATRT

**Fig. 3:**
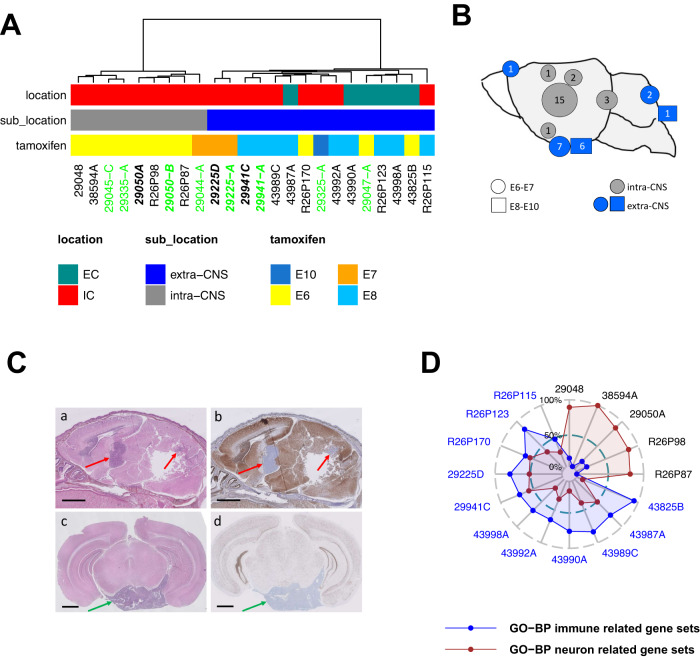
Genetically engineered mouse models reveal specific anatomical origins for murine Myc and Shh ATRT. **A** Unsupervised hierarchical clustering of mouse RT (*n* = 21 biologically independent mice) based on transcriptomic dataset of combined RNAseq dataset (*n* = 16 biologically independent samples, sample name colored in black) and gene expression microarray dataset (*n* = 8 biologically independent samples, sample name colored in green). 3 samples (bold sample names) were processed using the two platforms and used to control the cross-platform data integration. **B** Anatomical distribution of Rosa26::CreERT2/Smarcb1flox/flox (R26) RT; circles correspond to Tamoxifen injection at E6-E7 while squares correspond to E8-E10; gray indicates intra-CNS tumors, blue, indicates extra-CNS tumors. **C** Hematoxylin Eosin Safran (HES, a, c) and BAF47 staining (b, d) of sagital (a, b) and coronal (c, d) sections of brains from the R26 mice. Red arrows point to intra-CNS tumors (a, b); green arrows point to extra-CNS tumors (c, d). Scale bars: 1 mm (**D**) Single sample gene set enrichment analysis of murine RT samples based on RNAseq data (*n* = 16). Each dot corresponds to one sample; sample names are colored according to molecular subgroups (black: Shh; blue: Myc). Average enrichment scores (in percentage) of the top 5 differentially enriched neuronal development-related (red) and immune-related (blue) gene sets are considered and indicated for each sample. Source data are provided as a Source Data file.

We next aimed to investigate how these four anatomical-molecular subgroups, based on anatomic location and molecular profiling, were also relevant in our previously published mouse model of ATRT. We developed a mouse model (Rosa26-Cre^ERT2^::Smarcb1^flox/flox^) which, by an inducible inactivation of *Smarcb1* in unrestricted cell types, gives rise to Myc and Shh subtypes of ATRT^7^(Supplementary Data 3). To obtain more robust correlations between tumor molecular subtypes and anatomical sites of origin, we generated an increased number of tumors and profiled them by RNAseq and gene expression microarray. Using unsupervised hierarchical clustering on these datasets, we first confirmed the two Shh and Myc subgroups previously described. We thereby found that intracranial Myc clustered with the extracranial tumors (Fig. 3A and Supplementary Fig. 3A, B), in line with aforementioned observations in humans relating ECRT to MYC ATRT^5^. This corroborated the hypothesis that, in mice as in humans, Myc ATRT and extra-cranial tumors share certain molecular similarities, potentially revealing similar non-neuronal lineages of origin. Furthermore, a careful description of tumor locations clearly demonstrated that Myc intracranial tumors were in fact of extra-CNS origin, arising from the periphery of the brain, in meningeal spaces (Figs. 3A, B, C c, d; Supplementary Fig. 3A and 3B). These results fit well with the extra-axial origin and peripheral location of some human MYC ATRTs, as deduced from diagnostic magnetic resonance imaging (MRI) (Supplementary Fig. 1B), strengthening the hypothesis of a non-neuronal origin for this subgroup.

In contrast, all murine Shh ATRTs developed within the brain parenchyma (Fig. 3A); more precisely, 15/22 tumors were localized in the ventral sub-ventricular region, and invaded the basal ganglia (Fig. 3B, C a, b, Supplementary Fig. 3C: a, b), a location highly consistent with the description of human BG/IV SHH ATRT. In addition, we occasionally found tumor-like Smarcb1-deficient areas at the junction of the midbrain and the posterior fossa (Fig. 3C b, Supplementary Fig. 3C: c, d), which never reached the bulky volume observed in the basal ganglia region. This indicated that, although the CAL SHH ATRT was not properly recapitulated in this model, the corresponding region in mice allowed some abnormal proliferation of Smarcb1-deficient neurons. Of note, Shh/intra-brain parenchyma (intra-CNS) tumors were exclusively obtained after the earliest inactivation of *Smarcb1*, i.e. E6 to E7; conversely, Myc/extra-brain parenchyma (extra-CNS) tumors were obtained within a broader developmental window (E6 to E10) (Fig. 3A, B). This suggested earlier and more time-restricted progenitors for Shh ATRTs than for Myc ATRTs. In addition, the single sample Gene set Enrichment Analysis (GSEA) definitively pointed to a neuronal origin for Shh, whereas the Myc tumors were mainly characterized by an immune signature (Fig. 3D), as previously described^13^. These results supported the hypothesis that mice Shh ATRT and supratentorial human SHH ATRTs (BG/IV SHH ATRT) develop from similar anatomical structures.

### BG/IV SHH ATRT and murine Shh ATRT show a unique expression pattern suggesting a ganglionic eminence origin

**Fig. 4:**
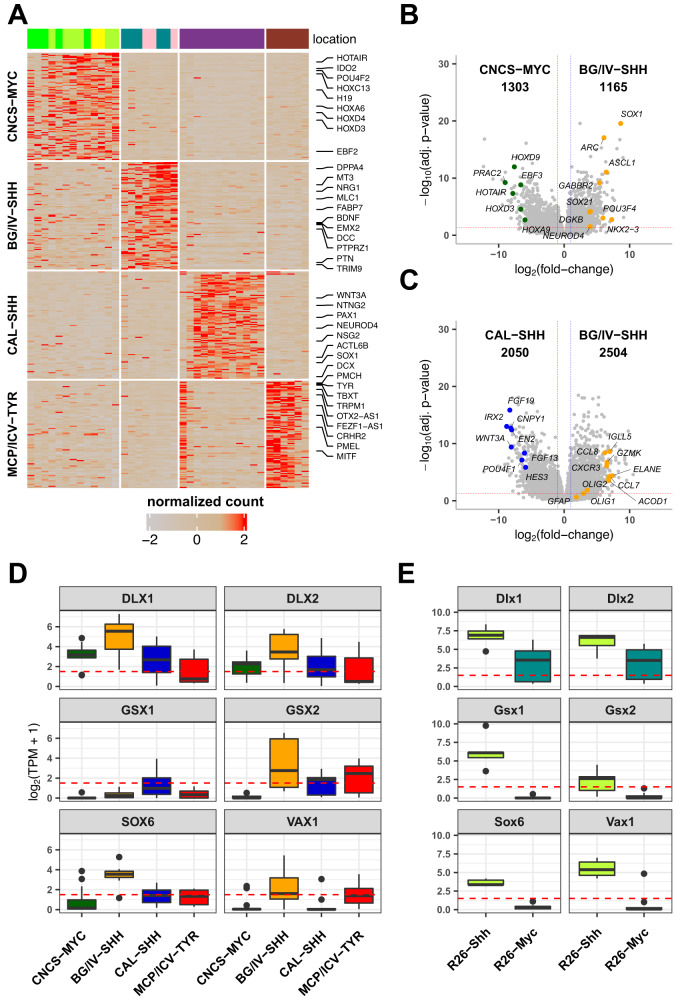
BG/IV SHH ATRT and murine Shh ATRT show a unique expression pattern suggesting a ganglionic eminence origin. **A** Heatmap of gene expression using the 100 most differentially expressed genes between anatomical-molecular subgroups in a “one versus all others” manner. Top annotation indicates sample anatomical location. Genes of interest are listed at the left of the heatmap; expression levels are ranked from the lowest (gray, −2) to the highest (red). **B**, **C** Volcano plots showing differential gene expression analysis results of BG/IV SHH versus MYC (b) and BG/IV SHH versus CAL SHH (c). The *x* axis indicates the log2 transformed fold-change and the *y* axis indicates the reverse of the log10 transformed adjusted *p*-value. Horizontal red line corresponds to adjusted *p*-value equals to 0.05 and two vertical blue lines indicate log2(fold-change) respectively equal to = −1 (left) and 1 (right). Differentially expressed genes of interest are labeled. Negative binomial GLM and Wald test were applied for gene expression comparison and generated *p*-values were corrected using the Benjamini and Hochberg method. **D**, **E** Boxplots of ganglionic eminence gene expression in (**A**) human ATRT anatomical molecular subgroups (*n* = 39 total of independent samples: *n*_CNCS-MYC_ = 13, _nBG/IV-SHH_ = 8, *n*_CAL-SHH_ = 12, *n*_MCP/ICV-TYR_ = 6) and in (**B**) mouse RT subgroups (*n* = 16 total of independant samples: *n*_*R26-SHH*_ = 5, *n*_*R26-MYC*_ = 11). *x* axis indicates subgroups and *y* axis indicates the level of expression in log2(TPM + 1). The box part of the boxplots represents the interquartile range while the whisker bonds of the boxplots indicate the highest and smallest values within 1.5 times interquartile range above and below the 75th and 25th quantiles respectively. Source data are provided as a Source Data file.

Since we were confident that BG/IV SHH ATRT formed a distinct group from CAL SHH ATRT and shared similar location with Shh mice tumors, we addressed the specific gene expression signatures in both species. In humans, we compared the four anatomically and molecularly defined subgroups. When compared with all other groups, BG/IV SHH ATRT were characterized by the overexpression of genes involved in forebrain development (*FOXG1*, *EMX2*, *ARX*, and *NRG1*), neurogenesis, synapse and neuronal plasticity (*ARC*, *LRRTM3* and *BDNF*), glial markers (*FABP7*, *OLIG2*, *GFAP*, *MLC1*) and pluripotency genes (*DPPA4*) (Fig. 4A, Supplementary Fig. 4G, Supplementary Data 4). When compared with MYC ATRT only, BG/IV SHH ATRT again showed a neuronal signature (Fig. 4A, B, D; Supplementary Fig. 4A, B, D; Supplementary Data 5). In contrast, when compared with CAL SHH ATRT only (Supplementary Fig. 4B, C), BG/IV SHH ATRTs were characterized by genes related to immune response (Fig. 4C; Supplementary Fig. 4F; Supplementary Data 6). Using deconvolution and immune infiltration estimation methods on both transcriptomic and DNA methylation datasets, we found that BG/IV SHH ATRT shared with MYC ATRT a more prominent immune infiltrate than TYR and CAL SHH ATRT, which likely accounted for their «MYC-like» expression signature (Supplementary Fig. 4H–O). However, the immune response observed in MYC ATRT and BG/IV SHH ATRT showed some differences such as a higher regulatory T cell infiltrate in MYC ATRT and a higher level of NK cells in BG/IV SHH ATRT (Supplementary Fig. 4N, O). These results were confirmed by immunostaining using CD3, CD8, and CD163, showing a more prominent immune infiltrate in BG/IV SHH ATRT than in CAL SHH ATRT (Supplementary Fig. 4P, Q).

Besides genes related to immune response, the top-expressed genes in BG/IV SHH ATRT contained the typical core set of genes expressed in the ganglionic eminence (Fig. 4D). The ganglionic eminence is a transitional structure with a key role during forebrain development; it is a progenitor domain that gives rise to cortical interneurons and, eventually, basal ganglia^14,15^. The expression of the ganglionic eminence core set of genes is consistent with the anatomic location of BG/IV SHH ATRT and suggested that cells from the ganglionic eminence could be at the developmental origin of this subgroup.

To investigate how mouse Shh ATRT actually recapitulates human BG/IV SHH ATRT, we first performed an unsupervised hierarchical clustering pooling mouse and human dataset. This showed that Shh mouse tumors clustered with CAL SHH ATRT rather than with BG/IV SHH ATRT, despite their clearly distinct locations (Supplementary Fig. 5A). However, considering that the immune infiltrate noticeably influenced the transcriptome-based clustering of BG/IV with MYC ATRT, despite their clearly neuronal features, we hypothesized that the difference between mouse Shh ATRT and BG/IV ATRT could be related to the influence of micro-environment rather than pointing to different origins. We therefore considered that the cell identity could be also assessed by the analysis of specific transcription factors (TF), known to play a critical role in neuronal lineages’ differentiation. In that line, ganglionic eminence marker genes that characterized BG/IV ATRT were also clearly overexpressed in the mouse Shh tumors (Fig. 4E), while TF found in CAL were much less clearly overexpressed (Supplementary Fig. 5B). Altogether, this shared TF expression in tumors localized in similar brain regions (Fig. 3C a, b) still suggested relevant homologies between Shh mouse and BG/IV ATRT. Together with the single sample GSEA pointing to embryonic brain neuron signatures (Fig. 3D), these results suggested neural progenitors from the ganglionic eminence as putative candidate cells of origin for basal ganglia Shh ATRT.

### Single-cell RNAseq suggests ganglionic eminence neural progenitors as putative cells of origin of BG/IV-ATRT

**Fig. 5:**
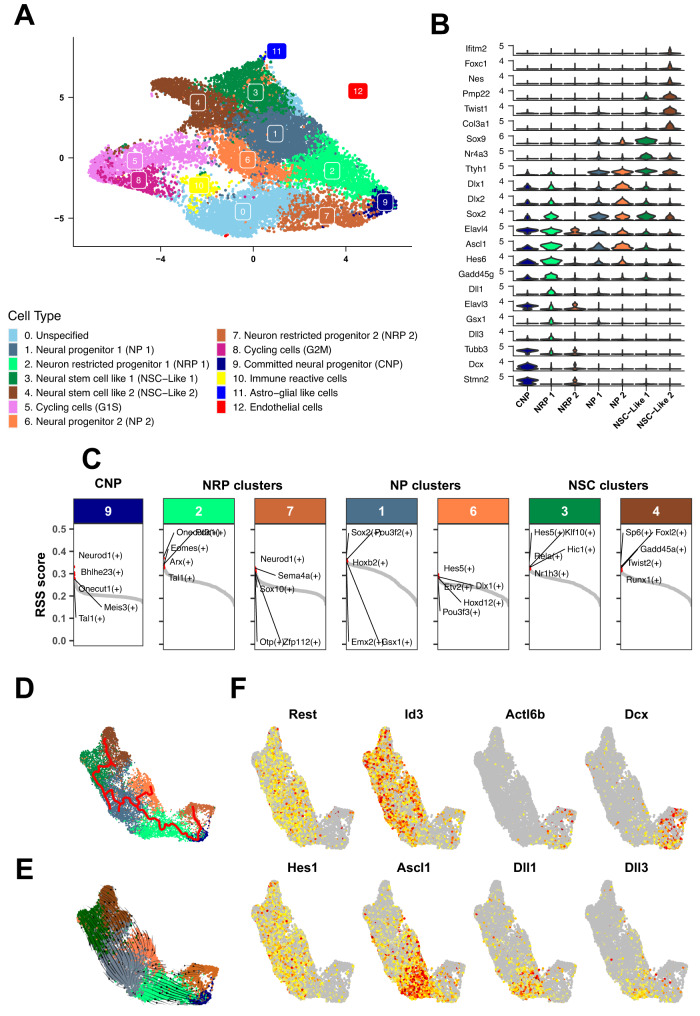
Single-cell RNAseq suggests ganglionic eminence neural progenitors as putative cells of origin of BG/IV-ATRT. **A** UMAP visualization of the mouse Shh single cell clusters obtained after the integration of 3 independant samples. Colors distinguish the different clusters; assigned names are reported at the right of the UMAP. **B** Violin plots showing the specific marker genes for neuronal clusters. **C** Regulon specificity score (RSS) for each transcription factor (TF) in Neuronal like clusters. In each cluster, regulons (TF along with their direct targets) are ordered according to their RSS. Interesting regulons are labeled. **D** Trajectory inference analysis using the PAGA algorithm (Monocle3). Colors distinguish the different clusters as reported in the legend of (**A**). **E** Embedding streams (RNA velocity – scVelo) showing the transcriptional dynamics throughout the trajectory. Colors distinguish the different clusters as reported in the legend of (**A**). **F** Heatmap showing the gradual expression along the trajectory of: the Notch signaling (*Hes, Dlk, Ascl1*), the SWI/SNF subunit (*Actl6B*) and neuronal differentiation gene (*Rest, Id, Dcx*) expression. Source data are provided as a Source Data file.

Taking advantage of the similarity between anatomical location and transcription factor modules of BG/IV and murine Shh ATRT, we assumed that single-cell transcriptomic analysis on mice tumors could further inform on the putative cells of origin in both species. We therefore performed scRNAseq on three murine Shh basal ganglia ATRT. We first checked the expression of genes that were known to be specific to SHH ATRT molecular subgroup identified in previous studies^2,3^; as expected, most of the genes related to the SHH ATRT signature were not homogeneously expressed, demonstrating the transcriptional intratumoral heterogeneity (Supplementary Fig. 6A). Then, our clustering approach suggested 13 different cell populations (Fig. 5A), for which marker genes were identified by differential expression analyses (Supplementary Data 7). Based on these marker genes, gene expression atlas databases (see Methods), as well as literature curation, we were able to infer the biological identity of each cell population (Fig. 5A, B; Supplementary Fig. 6B). In addition, in order to identify potential master regulator candidates for each cell population, we applied gene regulatory network (GRN) analysis using the SCENIC framework^16^, leading to the identification of transcription factors (TFs) characteristic for each cluster.

We first identified five clusters marked by non-neuronal marked genes: clusters 5 and 8 were characterized by the expression of genes of G1 to S and G2 to M cell cycle transitions, cluster 10 by immune response genes (*C1qa*, *Fcer1g*), cluster 11 by astro-glial gene markers (*Gfap, Aqp4*) and finally cluster 12 by endothelial gene markers (*Rassf9*, *Tm4sf1*) (Supplementary Fig. 6B). Interestingly, all the other clusters shared a subset of markers from the neuronal lineage (Fig. 5B, C). These clusters could be gathered in three main subgroups: (i) clusters 1 and 6 (“neural progenitor 1” and “neural progenitor 2”) characterized by the expression of neuronal progenitor markers (*Sox2, Dlx1, Dlx2*) and the activation of typical ganglionic eminence TFs (Cluster 1: *Gsx1*; Cluster 6: *Dlx1*); (ii) clusters 2, 7 and 9 (“neuron restricted progenitors 1”, “neuron restricted progenitors 2” and “committed neuronal progenitors”) characterized by the expression of neuronal differentiation genes (*Dcx*, *Stmn2* and *Tubb3*) and regulated by the transcription factors *Neurod1* and *Pou4f1;* (iii) clusters 3 and 4 (“neural stem cell like 1” and “neural stem cell like 2”), both characterized by neural stem cells markers (cluster 4: *Sox2*, *Sox9*, *Ttyh1*; cluster 3: *Sox2*, *Nes*) and a large variety of “non-neuronal” markers (Fig. 5B). Finally, cluster 0 fell apart from all others since it was massively characterized by non-coding RNAs, and the absence of marker genes of any specific cell type; it was therefore further referred to as “unspecified” cluster (Supplementary Data 7).

Aiming to identify the source of this cell diversity, we next performed trajectory inference analysis on the “neuronal” clusters (clusters 1, 2, 3, 4, 6, 7, and 9), using two different algorithms, the PAGA algorithm implemented in Monocle3^17^ and the elastic principal graph algorithm implemented in ElPiGraph^18^. In addition, RNA velocity analysis based on dynamical modeling^19^ was used to identify the root of the trajectory. These analyses suggested that the tumor cell populations studied here originated from the “neural progenitors 1 and 2” (Fig. 5D, E). These clusters were characterized by the expression of repressors of the terminal neuronal differentiation (*Rest* and *Id* genes) and the ganglionic eminence markers (*Ascl1*, *Dlx1*, *Dlx2*) (Fig. 5B, F). These cells then followed two antagonistic paths: (i) one towards the more committed neurons (clusters 7, 2, 9), which lost the neuronal repressors *Rest* and *Id* and switched to the post-mitotic *Actl6b* Swi/Snf member (Fig. 5D–F) and (ii) the other to the less differentiated cells, still characterized by neuronal lineage markers such as *Sox2* and *Sox9*, but also expressing more pleiotropic markers of mesodermal lineages (*Pmp22*, *Twist*) (Fig. 5B, F). Altogether, this trajectory analysis, in agreement with our hypothesis based on anatomic location and bulk RNAseq in both species, suggested that the murine Shh ATRT, and therefore possibly human BG/IV SHH ATRT, arise from neural progenitors from the ganglionic eminence, a subset of which will still be capable of neuronal commitment while another subset will dedifferentiate in more pleiotropic cells.

### CAL SHH ATRT anatomical location and molecular profile suggest a neuronal progenitor from the midbrain-hindbrain boundary as cell of origin

**Fig. 6:**
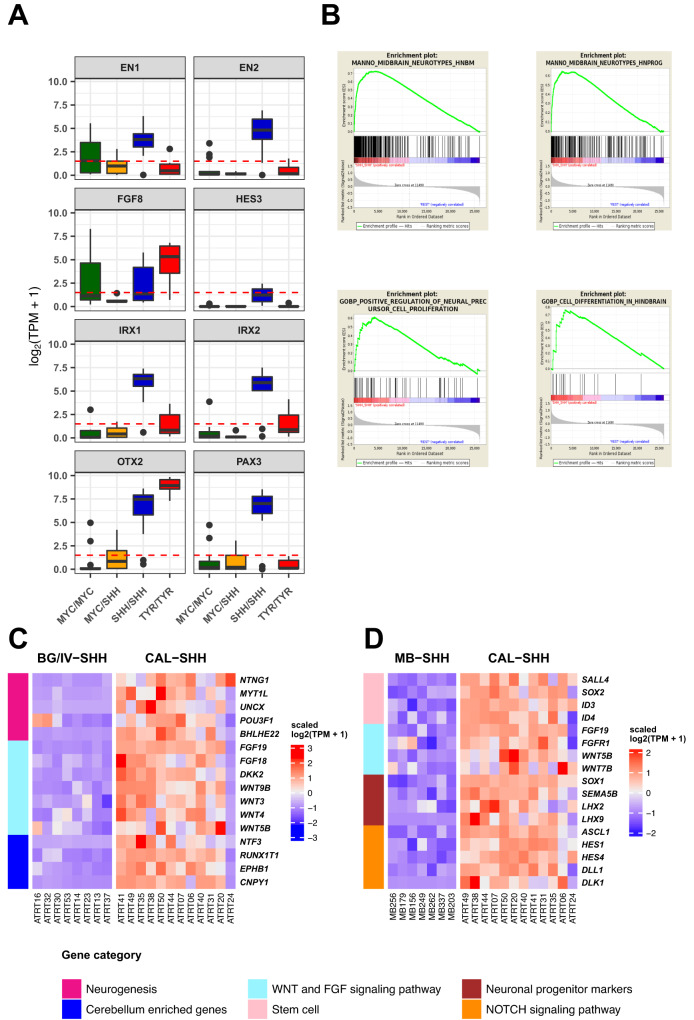
CAL SHH ATRT anatomical location and molecular profile suggest a neuronal progenitor from the midbrain-hindbrain boundary as cell of origin. **A** Boxplot showing the expression levels of genes involved in midbrain-hindbrain boundary (MHB) patterning in the human ATRT anatomical molecular subgroups (*n* = 39 total of independent samples: *n*_CNCS-MYC_ = 13, _nBG/IV-SHH_ = 8, *n*_CAL-SHH_ = 12, *n*_MCP/ICV-TYR_ = 6). *x* axis indicates subgroups and *y* axis indicates the expression level in log2(TPM + 1). The box part of the boxplots represents the interquartile range while the whisker bonds of the boxplots indicate the highest and smallest values within 1.5 times interquartile range above and below the 75th and 25th quantiles respectively. **B** Enrichment plot of midbrain/hindbrain gene sets in CAL-SHH resulting from GSEA between CAL SHH versus all other subgroups. **C**, **D** Heatmap showing the level of expression of a selection of genes differentially expressed in CAL SHH (*n* = 12) compared to BG/IV SHH (*n* = 8) (c) and to medulloblastoma SHH subgroup (*n* = 7) (d). Color codes at the right of the heatmaps refer to biological functions that are depicted below figures **C** and **D** Source data are provided as a Source Data file.

Since we felt confident that CAL SHH ATRT formed a distinct group, we sought to identify its specific transcriptomic profile as we did for BG/IV SHH ATRT. Differential gene expression analysis between CAL SHH ATRT and all other subgroups identified the over-expression of genes related to neurogenesis and neuronal migration (*SOX1, NTNG2, NEUROD4, NSG2, DCX*), WNT and FGF signaling pathways (Fig. 4A, Supplementary Data 4) and genes involved in midbrain-hindbrain boundary (MHB) patterning and cerebellum development (Fig. 6A)^20–24^. The MHB is an embryonic region delimiting the midbrain and the hindbrain and organizing the fate of neuronal progenitors from both sides of the edge, an embryonic structure of particular interest given the location of CAL SHH ATRT at the edge of supra- and infratentorial regions. This region is specified by the expression of typical genes and signaling, such as *FGF8* and *WNT3A* at the edge, markers of the rostral and caudal structures (Iroquois homeobox genes (*IRX1*, *IRX2*) and Engrailed family (*EN1, EN2*)), *HES3* and *PAX3*^25–31^. All these MHB core genes were characteristic for CAL SHH ATRTs (Fig. 6A), while GSEA pointed to embryonic neuronal development and midbrain or hindbrain patterning gene sets (Fig. 6B).

Focusing on differential expression analysis between CAL and BG/IV SHH ATRT only, we confirmed that CAL SHH ATRT were characterized by WNT and FGF signaling and MHB signature (Figs. 4C and 6C). Finally, considering that CAL SHH ATRT and infant SHH medulloblastomas develop in the cerebellum of young children and activate the SHH pathway, we assumed that a comparison of gene expression of these two types of tumors could also be relevant on the actual identity of CAL SHH ATRT (Supplementary Fig. 7; Supplementary Data 8). The MHB signature characterized again the CAL SHH ATRTs, strengthening our previous results. In addition, compared to SHH medulloblastomas, CAL SHH ATRT also showed the overexpression of genes involved in *NOTCH*, *WNT,* and *FGF* signaling pathways and the overexpression of stem cell and neuronal progenitor genes markers (Fig. 6D).

### Single-cell RNAseq analysis reveals transcriptional intra-tumoral heterogeneity of cerebellar anterior lobe ATRTs and neuronal progenitors as putative cells of origin

**Fig. 7:**
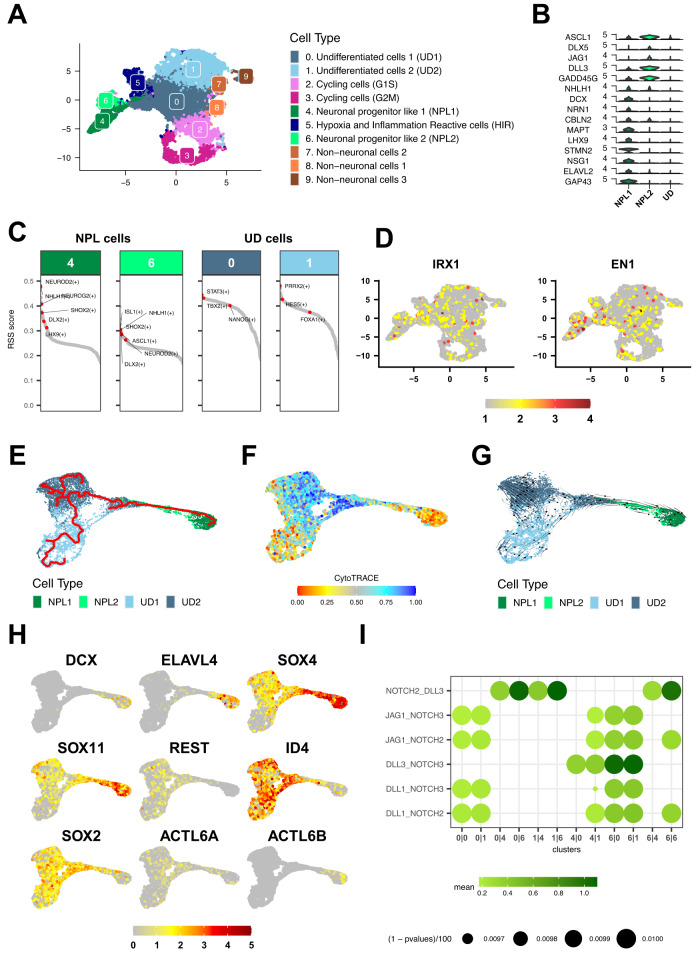
Single-cell RNAseq analysis reveals transcriptional intra-tumoral heterogeneity of cerebellar anterior lobe ATRTs and neuronal progenitors as putative cells of origin. **A** UMAP visualization of the human CAL SHH cell clusters obtained after integration 4 independent samples. Colors distinguish the different clusters; assigned names are reported at the right of the UMAP. **B** Violin plots showing the specific marker genes for Neuronal progenitor like cells and undifferentiated cells. **C** Heatmap of Midbrain/Hindbrain boundary gene signatures (*IRX1*, *EN1*) expression on the UMAP of integrated human CAL-SHH ATRT samples. **D** Regulon specificity score (RSS) for each transcription factors (TF) in Neuronal progenitor and undifferentiated cell populations. In each cluster, regulons (TF along with their direct targets) are ordered according to their RSS. Regulons of interest are labeled. **E** Trajectory inference analysis using the PAGA algorithm (Monocle3) showing a path form NPL1 cluster to UD clusters via NPL2 cells. Color code indicates cell clusters as referred in (**A**). Dark green: NPL1 cells, light green: NPL2 cells, light blue: undifferentiated cells. **F** Heatmap of CytoTRACE score at single cell level. The color gradient indicates a differentiated state (red) to an undifferentiated state (blue). **G** Embedding streams (RNA velocity – scVelo) showing the transcriptional dynamics throughout the trajectory. Genes specifically expressed in NPL1, NPL2, UD1, and UD2 cell clusters were used. Dark green: NPL1 cells, light green: NPL2 cells, light blue: undifferentiated cells. **H** Heatmap of gene expression showing the gradual expression along the trajectory of neurogenic TFs (*SOX4*, *SOX11*), neuronal differentiation genes (*DCX*, *ELAVL4*), neuronal repressor (*REST)*, stem cell and pluripotency markers (*ID4*, *SOX2*) and the SWI/SNF subunits genes (*ACTL6A*, *ACTL6B*). The color gradient indicates the expression levels, from the lowest (gray) to the highest (red). **I** Dot plot of NOTCH signaling ligand-receptor interaction between two cell clusters. Color gradient indicates the average expression of the ligand-receptor partner in the two clusters while the diameter of the dot indicates the corresponding *p*-value. Source data are provided as a Source Data file.

As aforementioned, we failed to obtain bulky tumors from the midbrain/hindbrain boundary that would recapitulate the CAL SHH ATRT subgroup in the Rosa26-Cre^ERT2^::Smarcb1^flox/flox^ model. Thus, to get further insights on putative cells of origin of the CAL SHH ATRT, we performed scRNAseq on four fresh human tumor specimens. As expected, the expression of typical SHH ATRT genes identified in Johann et al.^2^ and Torchia et al.^3^ was found heterogeneously among cells, suggesting the transcriptional intra tumoral heterogeneity of CAL SHH ATRT (Supplementary Fig. 8A). Different clustering approaches were applied (see Methods) leading to the identification of 10 clusters (Fig. 7A). Aiming to determine the biological identity of each cluster, we performed differential expression analyses (Supplementary Data 9) as well as GRN and identified marker genes and activated TFs characteristic for each cluster, as described above for mice Shh ATRT. Using atlas databases (see Methods) and literature review, we assigned a biological identity to each of the identified clusters (Fig. 7A, B). We first isolated two cycling clusters corresponding to G1 to S and G2 to M transitions (clusters 2 and 3, Supplementary Fig. 8B). We next found two clusters (clusters 4 and 6) characterized by the expression of different neuronal markers such as *GADD45G, NHLH1, DCX, NRN1, CBLN2,* and *MAPT*^6,32–37^. These two clusters also showed the activation of the same neurogenic TFs such as *ISL1*, *NHLH1*, *NEUROD2*, *DLX2* and *SHOX2*, suggesting that they were closely related (GRN analysis, Fig. 7C). In addition, cluster 4 presented specific expression of *LHX9*, *GAP43* and *ELAV2* genes, related to neuronal differentiation, whereas cluster 6 showed a specific expression of *ASCL1*, *JAG1*, *DLL3*, *DLX5* and *NEUROG1*, also suggesting a neuronal commitment but with a prominent NOTCH pathway activation and a less differentiated state than cluster 4 (Fig. 7B). Of note, the MHB marker genes identified from bulk RNAseq analyses (*IRX1*, *EN1*) were found expressed in these two clusters, although not restrictively (Fig. 7D). These two clusters were then referred to as “Neuronal Progenitor-like 1” (cluster 4) and “Neuronal Progenitor like 2” (cluster 6). In addition, we identified three clusters characterized by the expression of endothelial and glial genes markers (clusters 7, 8 and 9) that were referred to as “non-neuronal” clusters (Fig. 7A, Supplementary Fig. 8B, C) and one cluster characterized by the expression of genes involved in the hypoxic inflammatory response (cluster 5, Supplementary Fig. 8B). Finally, clusters 0 and 1 didn’t show any marker genes specific of known normal cell types, (Fig. 7B, Supplementary Data 9), which lead us to assign them as “undifferentiated”. However, they showed activation of TFs related to stem cell and pluripotency maintenance (*NANOG*^38–40^), epithelial-mesenchymal transition, and mesodermal commitment pathway (*PRRX2, MEOX1, FOXA1,* and *TBX2*)^41^, and neuroglial fate determination (*SOX9*)^42,43^, indicating some stemness features (Fig. 7C). The exclusive expression of stemness-related (*OTX2*) and neuronal committed-related markers (*DCX*) were confirmed by immunostaining (Supplementary Fig. 8D).

To investigate the relationship between the different clusters showing neuronal or stem cell features, we performed trajectory inference analyses on the “Neuronal progenitor like” and the “Undifferentiated” clusters, using similar approaches as described for murine Shh ATRT. These approaches gave consistent results and identified a path linking “Neuronal progenitor” clusters to the “undifferentiated” clusters (Fig. 7E, Supplementary Fig. 8E). Trajectory inference analysis using CytoTRACE^44^ shows gradual change on cell differentiation state throughout the trajectory, suggesting differentiation or dedifferenciation processes involved in the tumor development (Fig. 7F). RNA velocity analysis showed that the “Neuronal progenitors” from cluster 4 was the origin of the “undifferentiated” cells (Fig. 7G), while pseudotime analyses highlighted a consistent temporal progression, starting from “Neuronal progenitors” from cluster 4 and leading to the “undifferentiated” cells (Supplementary Fig. 8F).

### Notch pathway plays a role in the differentiation blockade

To understand the molecular mechanisms underlying this transition from “Neuronal progenitors” to the “Undifferentiated” cell clusters, we explored the expression of genes throughout the trajectory. Interestingly, we found a clear split between clusters expressing repressors of neuronal differentiation (*REST* and *ID* genes)^45–47^, neural stem cell markers (*SOX2*)^31,48^ and *ACTL6A*, and clusters expressing markers of neuronal differentiation (*DCX*, *ACTL6B*) (Fig. 7H). Similarly, we observed a clear separation between clusters expressing NOTCH pathway ligands, and clusters expressing NOTCH receptors and intracellular signaling actors (Supplementary Fig. 8G), suggesting a cross-talk between clusters via the NOTCH pathway. To confirm this hypothesis, we performed ligand-receptor interaction analysis using the CellPhoneDB framework^49^ and found that the ligands of NOTCH signaling were specifically expressed in the neuronal progenitor clusters, while the NOTCH receptors were specifically expressed in undifferentiated clusters (Fig. 7I). Altogether, these results suggested that CAL SHH ATRT emerge from neuronal progenitors at the MHB, that, following *SMARCB1* inactivation, mostly dedifferentiate by promoting neuronal program repressors.

Interestingly, re-analysing the only single cell sequencing of CAL SHH ATRT sample available in the literature (Jessa et al.^6^, sample ATRT5), we also clearly found that genes related to undifferentiated states (*SOX2*, *FABP7*, *OTX2*, *ID4, TTYH1*) distinguished the most prominent clusters from a minor population expressing more committed markers (*STMN4, DCX*), findings that corroborate our observations on an independent dataset (Supplementary Fig. 9A). Again, genes involved in the NOTCH pathway (*HES5*, *DLL3*) distinguished these clusters.

**Fig. 8:**
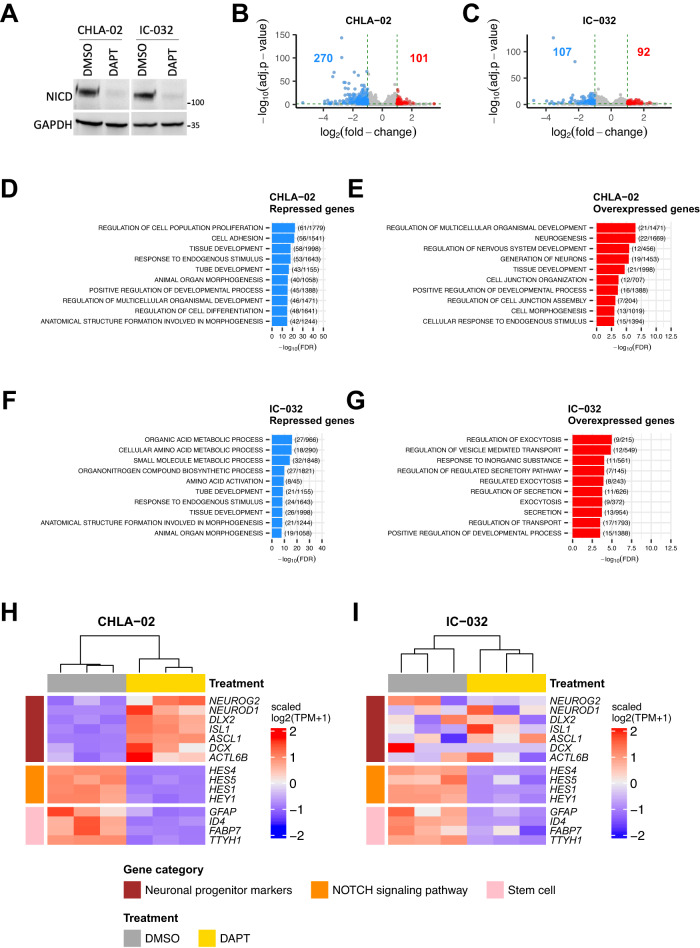
Inhibition of NOTCH signaling in rhabdoid cell lines. **A** Western blot analyses for Notch intracellular domain NICD in CHLA-02 and IC-032 cell lines treated with DAPT NOTCH inhibitor or DMSO as a control. The experiment has been repeated 2 times for CHLA-02. **B**, **C** Volcano plot showing the differential gene expression analysis results of DAPT treatment versus DMSO in CHLA-02 (**B**) and in IC-032 (**C**) cell lines. The *X* axis indicates the log_2_ transformed fold-change and the *Y* axis indicates the reverse of the log_10_ transformed adjusted *p*-value. The number of significantly repressed and overexpressed genes are labeled in blue and in red, respectively. Genes having an absolute fold-change higher than 2 and an adjusted *p*-value lower than 0.05 are colored either in blue (for genes repressed in DAPT-treated cells) or in red (for genes overexpressed in DAPT-treated cells). The dotted green horizontal and vertical lines correspond to a *p*-value = 0.05 and to absolute fold-change = 2, respectively. Negative binomial GLM and Wald test were applied for gene expression comparison and generated *p*-values were corrected using the Benjamini and Hochberg method. **D**, **E** Top ten significantly enriched Gene Ontology (Biological Processes only) gene sets in genes that are differentially repressed (**D**) or overexpressed (**E**) in DAPT-treated CHLA-02 cells compared with DMSO. **F**, **G** Top ten significantly enriched Gene Ontology (Biological Processes only) gene sets in genes that are differentially repressed (**F**) or overexpressed (**G**) in DAPT-treated IC-032 cells compared with DMSO. **H**, **I** Heatmap showing the level of expression of a selection of interested genes in DAPT-treated cells compared with DMSO in CHLA-02 (**H**) and in IC-032 (**I**) cell lines. Color codes at the left and at the top of the heatmaps are shown below the figure. Source data are provided as a Source Data file.

Our results suggested that the activation of the NOTCH pathway could mimic a lateral inhibition process that is also known to play a role in repressing the normal neuronal differentiation^50–53^. To get an insight on how NOTCH pathway could participate to cell reprogrammation, we treated two SHH ATRT cell lines (CHLA-02, and IC-032) with DAPT, a gamma secretase inhibitor (Fig. 8A). Although the treatment did not significantly affect cell viability (Supplementary Fig. 9B), it clearly impacted gene expression (Fig. 8B, C, Supplementary Data 10–11). As assessed by gene ontology analyses, differentially expressed genes were involved in developmental and differentiation programs (Fig. 8D–G). NOTCH signaling actor genes (*HES5*, *HES1*, *HES4*…) were clearly repressed as well as stem markers (*TTYH1*, *GFAP*, *FABP7*, *ID4*) typical of SHH ATRT (Fig. 8H, I); in CHLA-02 at least, the treatment also induced neuronal progenitor markers (*ISL1*, *NEUROG2*, *NEUROD1*), findings consistent with an overall dysregulation of development-related gene ontologies.

## Discussion

To date, multiple studies in humans have attempted to correlate ATRT molecular subgroups with predominant supra or infra-tentorial anatomical location, failing to accurately correlate the anatomical site with the molecular profiles and to provide information about the putative lineage of origin of each ATRT subgroup^2–4,54,55^. Here, we have investigated by an integrative analysis whether the precise anatomical description could improve the understanding of ATRT development. This approach allowed us to identify four distinct anatomical-molecular subgroups, which in turn allowed us to investigate the putative lineage of origin. We first found that a significant subset of MYC ATRT emerge from extra-axial locations such as cranial nerves; this feature has been repeatedly described in previous case reports^56–62^ that we believe, from our own findings, could be related to the lineage of origin of MYC tumors. The location of Myc murine ATRT, arising outside of the brain in the Rosa26-Cre^ERT2^::Smarcb1^flox/flox^ model, corroborated the hypothesis of an extra-axial origin for this subtype of tumors. These findings are consistent with (i) the SMARCB1-related schwannomatosis, which tightly links *SMARCB1* to oncogenesis in mature peripheral nerves^63–66^ (ii) the previously published phylogenetic links between Schwann cells and ECRT, again indicating a possible neural-crest origin for rhabdoid tumors^8^, (iii) the unique clustering to which MYC ATRT and ECRT belong, already suggesting a common non brain origin for these types of rhabdoid tumors^5^, and (iv) the typically extra-axial origin of ATRTs in the P0-Cre::Smarcb1^flox/flox^ model, originating from the cranial nerves and the periphery of the brain, locations very similar to the ones we described for human MYC ATRT, and also pointing to neural-crest cell precursors as lineage of origin. In a recently published work, Graf et al. performed scRNAseq on a series of Myc murine ATRT, which was not done in the present manuscript^10^. Of note, those authors related this tumor subset to primordial germ cells based on correlation with atlases, a finding that does not fully fit with the aforementioned studies. Whether these results reveal some specificity for murine Myc tumors, some diversity among the potential origins of Myc tumors, or simply indicate that *SMARCB1* abrogation can, in some contexts, erase all specific preexisting gene expression in such a deep manner that the transcriptome matches with primordial germ cells, remains to be further demonstrated. Further work is therefore needed on human and mouse MYC ATRT to fully elucidate this enigma.

Our study also established that SHH ATRT, canonically defined using DNA methylation profiling, are in fact composed by at least two anatomical-molecular subgroups. While we were preparing this manuscript, Federico et al. published similar findings on an independent series of SHH ATRT^67^, depicting three subgroups, with clearly distinct locations and gene expression profiles. “ATRT SHH-2” in the Federico study overlaps with our CAL SHH ATRT regarding the location and the overexpression of genes such as *EN2*, supporting our results about the origin of these tumors in the midbrain hindbrain boundary. Moreover, “ATRT SHH-1A” largely corresponds to our BG/IV SHH ATRT, and, interestingly, overexpress *OLIG2*, a glial marker that we also found overexpressed in the bulk RNAseq analyses, together with several neuronal markers, again underscoring the multiple lineage markers characteristic for this group. Our series comprises less SHH supratentorial cases with more restricted age distribution (between 0.7 and 6.2); this fact may explain why we don’t identify the third subgroup recently described by Federico et al., which could be part of our BG/IV SHH ATRT. By including a comprehensive radiological review, our study helped orienting the interpretation of the bulk expression profiling and extends the results of Federico et al. by adding scRNAseq for two of these subgroups. This combined approach taking into account the precise anatomical location to interpret gene expression profiling allowed us to raise hypotheses for both CAL and BG/IV SHH ATRT, i.e., progenitors from the MHB and the ganglionic eminence, respectively. These hypotheses now need to be definitely confirmed using mouse models expressing Cre in specifically restricted progenitors.

One unexpected finding of our study was the immune signature distinguishing BG/IV from CAL SHH ATRT, that accounted for the discrepancy between methylation-based and gene expression-based classifications for this particular subgroup. Previous studies depicting the immune infiltrate showed partly discrepant results regarding SHH ATRT immune infiltrate^13,68^, which may be explained by the use of different subgrouping techniques. It also suggests that the abundance of the immune infiltration may be due to some anatomical constraints as much as to intrinsic tumor cell properties. Interestingly, the low immune infiltrate seen in CAL ATRT is to be compared to the similarly low immune infiltrate in mouse Shh ATRT. Altogether, this suggests that not only the SHH or MYC methylation profiling, linked to the neuronal versus non-neuronal features of ATRT, should be considered to predict potential efficacy of immunotherapies, but rather immunohistochemistry and/or precise subtype definition.

Since these neural progenitor regions play a crucial role in brain segmentation processes^69–72^, SHH ATRT could be considered as a disease of embryonic brain segmentation. Moreover, single cell transcriptomic approaches suggested that *SMARCB1* loss in the appropriate neural progenitors drives a dedifferentiation process through the induction of neuronal program repressors such as *REST* and *ID* genes, leading to the emergence of neural stem cell markers including *ACTL6A* and *SOX2*. In none of these SHH ATRT did the SHH signaling seem to play a major role in cluster distinction. In contrast, we clearly noted a prominent role of NOTCH signaling which has already been described to be characteristic for SHH ATRT^2,67^. Our results suggest that lateral inhibition involving the NOTCH pathway may play an important role in the inter-cluster cross-talks, and possibly in the dedifferentiation process, as suggested by the effects of NOTCH inhibition on SHH ATRT cell lines. Targeting NOTCH signaling would therefore deserve further explorations in an interceptive strategy for the treatment of both types of SHH ATRT.

## Methods

### Animals

Mouse strain Rosa26-Cre^ERT2^::Smarcb1^flox/flox^ (Han et al.^7^) was genotyped according to this reference. All experiments were performed on mixed background (129/SV×C57BL/6). The sex ratio within groups was in equilibrium. Protocol and animal housing were in accordance with national regulation and international guidelines^73^.

Approval for this study was received from the Institutional CEST review board (Comité d’Evaluation et de Suivi de Recherche Translationnelle) from Curie Institute, and from the Direction Generale de la Recherche et de l’Innovation, Ministere de l’Enseignement Superieur et de la Recherche (authorization number 6,150).

Tamoxifen administration to recombine Smarcb1 locus was performed according to the same reference. Mice were sacrificed according to the ethical approval and when neurological behaviors reached the ethical limit endpoints.

### Mouse tumor monitoring

Mice were monitored for tumor formation at least 3 times per week. The observation period encompassed at least 18 months. Intra-CNS tumors could not be measured. Mice weight was checked daily from any neurological symptom (weakness, circling, tremor, paralysis) and mice were sacrificed when weight loss reached 20%. For the rare extracranial tumor, mice were sacrificed when tumors reached 1000 mm^3^. Tumors and all organs were taken and fixed in AFA for histology or were frozen in −80 °C until RNA extraction. Three fresh tumors were processed for scRNA-seq.

### Mouse sample histological examination

Organs were collected, frozen on dry ice and processed for cryosectionning and macro-dissection or fixed in AFA (Carlo Erba, ref: 526263001) for histological examination. BAF47 immunohistochemistry was performed on fixed paraffin-embedded tissue using BD, code 612111, clone 25/BAF47, dilution 1/50^74^. Immunohistochemistry Ki67: primary antibody (sc-7846) incubation 1 h (dilution 1:300), secondary antibody anti-goat BIOT (705-006-147 Jackson) incubation 25 min (dilution 1:250), staining with Vectastin Kit.

### Mouse tumors macrodissection and RNA extraction

Frozen brains were serially sectioned using a cryostat at 4 mm; quick Hematoxilin stainings were performed on each section until a tumor could be identified; macrodissection was then performed with a sterile scalpel. Small pieces of tissue containing the tumor cells were frozen at −80 °C until RNA preparation. The tumor RNAs were extracted using a miRNeasy mini kit (Qiagen ref: 217004).

### ATRT Tumor samples

Freshly resected and snap-frozen human ATRT samples were collected following written informed consent of parents regarding tumor banking and use for research; approval of these consents was obtained by the internal review board from Curie Institute and Necker Hospital for Sick Children (Paris, France, IRB approved protocol number DC-2009-955).

### Immunohistochemical analyses

A representative section was selected for each case. Unstained 3-μm-thick slides of formalin-fixed, paraffin-embedded tissues were obtained and submitted for immunostaining. The primary antibodies employed included programmed death-ligand 1 (PD-L1) (1:100, clone E1L3N, Cell Signaling Technology, Beverly, USA), PD-1 (1:250, clone EPR4877(2), Epitomics, Cambridge, USA), CD3 (1:50, clone F7.2.38, Dako, Carpinteria, USA), CD4 (1:80, clone 4B12, Leica Biosystems, Wetzlar, Germany), CD8 (1:25, clone C8/144B, Thermo Scientific, Waltham, USA), CD45 (1:500, clone PD7/26 and 2B11, Dako, Carpinteria, USA), CD57 (1:40, clone HNK-1, BD Biosciences, Franklin Lakes, USA), CD68 (1:400, clone KP1, Dako, Carpinteria, USA), CD163 (1:50, clone IHC163, Diagomics, Blagnac, France), FOXP3 (1:50, clone 206D, BioLegend, USA), Granzyme B (ready to use, clone 11F1, Leica Biosystems, Wetzlar, Germany), OX40 (1:100, clone ACT-35, Thermo Scientific, Waltham, USA), OTX2 (1:600, clone 1H12C4B5, Thermo Fisher, Rockford, USA) and DCX (1:200, clone EPR19997, Abcam, Cambridge, United Kingdom). All slides were stained using previously optimized conditions including positive and negative controls (human placenta for PD-L1 and human tonsil for other markers). PD-L1 expression was evaluated in the tumor cells using H-score, which includes the percentage of positive cells showing membrane staining pattern (0–100) and intensity of the staining (0–3+), with a total score ranging from 0 to 300. All other immunemarkers were evaluated as density of cells (0: absent; 1: scarce; 2: moderate, and 3: diffuse), defined as the number of positive cells per area (1 mm^2^) regardless of the intensity. The final score for each marker was expressed as the average score of the five areas analyzed within the tumor region. The final scores for each marker from each patient were then transferred to a database for statistical analysis.

### Cell culture and viability assays

ATRT cell line CHLA-02-ATRT (#CRL-3020, ATCC) was cultured according to the manufacturer’s protocol. IC-032 cell line, established in Curie Institute from a supra-tentorial ATRT, was cultured in DMEM/F12 supplemented with 20 ng/mL FGF, 20 ng/mL EGF, and 1x B27 supplement (#15360284, Fisher Scientific). To assess the effect of NOTCH inhibition on cell viability, 8 × 10^3^ cells per well were plated in triplicate in 96-well plates and treated either with DMSO vehicle control or varying doses of DAPT gamma-secretase inhibitor (#HY-13027, MedChem Express). After 7 days of treatment, resazurin (Sigma-Aldrich) was added (20 μg/ml) to assess cell viability and cells were incubated for another 2–6 h, depending on the cell line. Fluorescence signals proportional to the number of cells were recorded in a FLUOstar Omega plate reader (BMG labtech SARL).

### Western blot analysis

For NOTCH inhibition validation, CHLA-02-ATRT and IC-032 cell lines were treated with DMSO or DAPT at 10 µM for 7 days. Cells were then washed once with cold PBS and scraped on ice with lysis buffer (20 mM Tris-HCl pH 7.5, 1 mM EDTA, 0.5% NP40, 120 mM NaCl) supplemented with protease inhibitors (Roche). After centrifugation and quantification, protein extracts were resolved by sodium dodecyl sulfate–polyacrylamide gel electrophoresis before transfer onto nitrocellulose membrane. Immunoblots were done with monoclonal rabbit cleaved Notch1 (#4147, Cell signaling) and HRP-conjugated GAPDH (#HRP-60004, proteintech) antibodies. Cleaved Notch1-blotted membrane was then incubated with an anti-rabbit immunoglobulin G horseradish peroxidase-coupled secondary antibody (1:3000, NA934; Amersham Biosciences). Proteins were detected by enhanced chemiluminescence (Biorad).

### Gene expression profiling

#### RNA-seq library preparation

Total RNAs were obtained from ATRT (*n* = 10) and mouse RT (*n* = 16) frozen samples using Qiagen QIAamp RNAeasy kit, according to the manufacturer’s procedures (Qiagen, Venlo, Netherlands). For cell lines, CHLA-02-ATRT and IC-032 cells were treated with DMSO or DAPT at 10 µM for 7 days. RNAs were extracted using the Nucleospin II kit (Macherey-Nagel). Experiments were performed in triplicate. The tumor cell content was visually estimated before RNA extractions. Barcoded Illumina compatible libraries were generated from 750 ng of total RNA for each sample using TruSeq Stranded mRNA Library Preparation Kit (Illumina, San Diego, California, U.S.,). Libraries were sequenced using the Illumina HiSeq 2000/2500 or illumina NovaSeq platforms in the 100 bp paired-end mode. FASTQ samples were generated after demultiplexing the resulting BCL files.

#### RNA-seq data processing

Raw data were processed using an in-house pipeline developed at the Institut Curie Bioinformatics Core Facility, following standard analysis in the field and available at https://github.com/bioinfo-pf-curie/RNA-seq. Briefly, read mapping and counting were performed using STAR version 2.5.3a aligner^75^. The human reference genome hg19 and the mouse reference genome mm10 were used. The 10 ATRT RNA-seq data were combined with 9 from Andrianteranagna et al.^76^ and 29 from Leruste et al.^13^ to form a cohort of 49 RNA-seq samples that were re-analyzed from FASTQ files using the same pipeline.

#### RNA-seq statistical analysis

For each of the human and mouse datasets, all genes having 0 counts in all samples were filtered out before subsequent analyses. Variance stabilization process was then applied using the *rlog()* function of DESeq2 version 1.30.1 (Love et al.^77^) Bioconductor package. Genes having rlog counts lower than 7.5 in 99 % or more of the samples were filtered out. Only the 5000 top variable genes (based on IQR) were kept for the unsupervised analyses.

Principal component analyses (PCA) were performed using the *prcomp()* function of the R base package *stats* on centered and scaled data. Hierarchical clustering analyses were conducted using the *Heatmap()* function of the *ComplexHeatmap* version 2.6.2 (Gu et al.^78^) Bioconductor package. Pearson correlation and Ward’s method were used respectively as similarity metric and linkage method. Consensus clustering analyses were conducted to estimate the stability of the number of clusters. They were performed with the *ConsensusClusterPlus* version 1.54.0 (Wilkerson and Hayes^79^) (Bioconductor package using the same metrics of similarity and linkage as set for the hierarchical clustering. All other settings were set by default except the *pFeature* that we set to 0.8. The *ComplexHeatmap* package was used to visualize the consensus clustering result.

The Uniform Manifold Approximation and Projection (UMAP)^80^ non-linear dimensionality reduction algorithm was applied for visualization purpose. UMAP analyses were performed using the *umap* (version 0.2.7.0) CRAN packages.

Sparse Partial Least Squares Discriminant Analysis (sPLS-DA)^81^ was conducted using the mixOmics framework version 6.14.1 (Rohart et al.^82^) Bioconductor package. The optimal number of component as well as the number of gene per component were determined by running the *perf()* and *tune.splsda()* functions using 3-fold cross-validation repeated 50 times. Finally, sPLS-DA analysis was run using the *splsda()* function using 3 components with respectively the 90, 100 and 50 previously selected genes (Supplementary Fig. 2B).

Differential gene expression analyses were performed using *DESeq()* function of the DESeq2 package using the filtered raw counts. Resulting *p*-values were corrected using the Benjamini and Hochberg method (*a.k.a*. FDR). Immune cells and stromal cells infiltration scores were computed using the ESTIMATE version 1.0.11^83^ R-Forge package, a marker-based single sample gene set enrichment method. Immune cells (T cell CD8+, T cell CD4+, T cell regulatory, NK cell) relative fraction were computed using deconvolution-based quanTIseq algorithm (Finotello et al., 2019; Sturm et al., 2019) implemented in the immunedeconv version 2.0.4 R Bioconductor package. Analyses were performed inside R environment (version 4.0.2).

Functional enrichment analyses on human dataset were performed using the web application available in https://www.gsea-msigdb.org/gsea/msigdb/annotate.jsp and the GSEA tool (version 2.2.3) on the GO:BP gene set collection (version 7.4). Functional enrichment analyses on mouse dataset were performed using the GSVA (version 1.38.2) bioconductor package and the msigdbr (version 7.4.1) CAN package.

#### Gene expression array data processing and analysis

Eight Smarcb1flox/flox;Rosa26-CreERT2 mouse primary RT affymetrix (MOE430 2.0 array type) samples from Han et al.^7^ were re-analyzed. Data were normalized using the RMA method implemented in the affy version 1.70.0 Bioconductor package with the custom Brain array CDF annotation packages version 23.0.0 (http://brainarray.mbni.med.umich.edu/Brainarray/Database/CustomCDF/23.0.0/entrezg.asp). Gene filtering was performed using the PCA based approach described in Lu et al. (2011) and implemented in the pvac version 1.40.0 Bioconductor package. Hierarchical clustering was performed using the ComplexHeatmap version 2.9.4 Bioconductor package. GSEA are applied using the GSEA software version 2.2.3 and the MSigDB database version 5.2 downloaded from https://www.gsea-msigdb.org/gsea/msigdb/. Data analyses were performed using R environment (version 4.1.1)

#### Mouse RNA-seq and gene expression array data integration

8 mouse RT affymetrix data and 16 mouse RT RNA-seq data were combined based at gene level. The two datasets were previously normalized, filtered and scaled separately before merging. Platform technical effect was assessed using PCA and corrected using linear model implemented in *comBat()* function of SVA version 3.40.0 Bioconductor R package. Hierarchical clustering was applied to the combined dataset using the ComplexHeatmap version 2.9.4 package and the 5000 most variable genes (based on IQR). Euclidean distance and Ward linkage method were used.

#### Human and Mouse gene expression data integration

Human and mouse RNA-seq data were merged based on the orthologous genes (16864) between the two species identified using biomaRt package version 2.48.3. Genes having an expression lower than a threshold equals to 6 regularized counts in more than ¾ of the samples were filtered out. Sample-wise correlation between human and mouse datasets using genes of interest was performed based on the Pearson’s method. Organism effect correction was applied using the *ComBat()* function of the SVA package version 3.40.0 before unsupervised clustering on the merged dataset. The 1000 most variable genes (based on IQR value) were kept for the hierarchical clustering performed on the merged dataset. The analyses were conducted within R environment (version 4.1.1).

### DNA methylation array

#### DNA methylation array data processing

Infinium methylationEPIC array data from 54 human ATRT samples were collected (30 were retrieved from et al.^13^) and processed using *RnBeads* package version 1.6.1 (Assenov et al., 2014) Bioconductor package. All samples and all probes were kept after quality control. Probe intensities were normalized using the *rnb.execute.normalization()* function with the “illumina” method. Probes outside CpG context (2991 probes), targeting single nucleotide polymorphisms (17369 probes) or targeting X and Y chromosomes (19457 probes) were all filtered out before subsequent analysis.

#### DNA methylation array statistical analysis

Beta-value at probe level were extracted using *meth()* function. For a given CpG site, beta-value is the ratio of signal from methylated probes relative to signal from both methylated and unmethylated probes. The 5,000 highest variable probes (based on beta-value IQR) among the retained probes (828,109) were selected for the unsupervised analyses. Hierarchical clustering samples was performed with the *Heatmap()* function of *ComplexHeatmap* package. Euclidean and 1-Pearson were used as distance metrics respectively for probes and samples clustering. Ward’s method was used as linkage criterion for both sample and probes clustering. The UMAP non-linear dimensionality reduction algorithm was applied for visualization purpose using the umap R package. Leucocytes infiltration scores were computed based on the leucocytes unmethylated probes (LUMP) identified by Aran et al.^84^. For a given sample, this score is calculated as 1 substracted by the mean LUMP beta-values divided by 0.85. Lymphocytes score was computed, for each sample, as the mean of the lymphoid-specific hypermethylated probes identified by Killian et al.^85^. Data processing and analyses were performed inside R environment (version 4.0.2).

### Human gene expression and DNA methylation data integration

Gene expression (RNA-seq) and DNA methylation (EPIC array) data (“early”) integration were performed using the kernel-based method implemented in *mixKernel* version 0.7 CRAN package^12^. The analysis was conducted on the 43 samples in which both RNA-seq and DNA methylation data are available. For RNA-seq dataset, the rlog count matrix including only the 2,000 most variable genes (based on IQR value) was used while for DNA methylation dataset, the beta-value matrix including only the 5000 most variable genes (based on IQR value) was considered. The kernel matrix of each dataset was computed using the *compute.kernel()* function with the *“linear”* kernel method. The two kernel matrices were combined using the *combine.kernels()* function with the *“full-UMKL”* option. PCA as well as UMAP were applied on the meta-kernel using respectively the *prcomp()* function of the R base package stats and the *umap* version 0.2.7.0 CRAN package. Alluvial diagrams were generated using the *alluvial* version 0.1-2 CRAN package. Data analysis was performed inside R environment (version 4.0.2).

### Single cell RNA sequencing (scRNA-seq)

#### Tissue processing and cell population enrichment for human samples

Fresh tumor samples were cut in small pieces then dissociated 30 min at 37 °C in CO_2_-independent medium (Gibco) + 0,4 g/l of human albumin (Vialebex) with Liberase TL (Roche) 150 mg/ml and DNase 1 (Sigma) 150 mcg/ml. Dissociated cells were then filtered with a 40 mm cell strainer, then washed and resuspended with C02-independent medium + 0,4 g/l of human albumin. Cells were then continuously maintained on ice or at 4 °C. In case of lot of blood cells, the Debris removal kit (Miltenyi Biotec) was used according to the manufacturer’s protocol. To enrich in tumoral cells (human samples) the Tumor Cell Isolation Kit (Miltenyi Biotec) was used according to the manufacturer’s protocol. Cells were then resuspended in PBS + BSA 0.04%. Samples were prepared for concentration of 800 cell/mcl. Tissues were processed within 2 h after tumor resection and loaded in 10x Chromium instrument within 4 h.

#### Preparation of single cell suspensions for mouse samples

Fresh tumor samples were cut in small pieces then dissociated 30 min at 37 °C in CO_2_-independent medium (Gibco) + 0,4 g/l of human albumin (Vialebex) with Liberase TL (Roche) 150 mg/ml and DNase 1 (Sigma) 150 mcg/ml. Dissociated cells were then filtered with a 100 mm cell strainer, then washed and resuspended with C02-independent medium + 0,4 g/l of human albumin. Cells were then continuously maintained on ice or at 4 °C. In case of lot of blood cells. Cells were then resuspended in PBS + BSA 0.04%. Samples were prepared for concentration of 800 cell/mcl. Tissues were processed within 2 h after tumor resection and loaded in 10x Chromium instrument within 4 h.

#### Single cell RNA sequencing

Sample preparations were loaded on a 10x Chromium instrument (10x Genomics) and libraries were prepared using a Single Cell 3’ Reagent Kit (V2 chemistry, 10X Genomics) according to the manufacturer’s protocol, targeting 1000 recovered cells per sample. Single cells were included and barcoded into droplets together with gel beads coated with unique barcodes, unique molecular identifiers (UMI), and poly(dT) sequences, followed by in droplet reverse transcription to generate barcoded full-length cDNA. cDNA was subsequently recovered from droplets, then cleaned up with Dynabead MyOne Silane Beads (Thermo Fisher Scientific), then amplified with the following protocol: 98 °C-3 min; 12x (98 °C-15s, 67 °C-20s, 72 °C-1 min); held at 4 °C. Amplified cDNA product was cleaned up using the SPRI select Reagent Kit (Beckman Coulter). Indexed libraries were constructed following these steps: 1. Fragmentation, end repair and A-tailing; 2. Size selection with SPRI select beads; 3. Adaptor ligation; 4. Post-ligation cleanup with SPRI select beads; 5. Sample index PCR and final cleanup with SPRI selects beads. Library and quality assessment were achieved using dsDNA High Sensitivity Assay Kit and Bioanalyzer Agilent System. Indexed libraries were tested for quality, equimolarly pooled and sequenced on an Illumina HiSeq2500 using paired-end 26 × 98 bp as sequencing mode, targeting at least 50,000 reads par cell.

#### scRNA-seq data pre-processing

Raw data generated by the seqencing Illumina HiSeq2500 platform were pre-processed using the cellranger (version 3.1.0, https://support.10xgenomics.com/single-cell-gene-expression/software/pipelines/latest/what-is-cell-ranger) including demultipexing, mapping with the hg19 or the mm10 reference genome, gene counting and aggregation steps.

#### scRNA-seq cell filtering

Single sample adaptive filtering strategy was applied to remove “bad cells” in both human and mouse samples. The number of detected genes and the percentage of mitochondrial RNA were considered as the filtering criteria. Cells with both a low number of genes and a high proportion of mitochondrial RNA were discarded. The threshold of the minimum number of detected genes was set as the 5th percentile of the distribution of the number of detected genes in all cells (Supplementary Figs. 10A and 12A). The thresholds of the maximum proportion of mitochondrial genes were set individually for each sample based on the visual inspection of the plot of the number of detected genes versus the percentage of mitochondrial gene (Supplementary Figs. 10B and 12B). In addition, cell clustering was performed in individual sample to check if some covariates such as the number UMIs per gene ration (UMIs/gene) drive the clustering of cells (Supplementary Fig. 10D). For IRT003 samples, two clusters (3 and 4, Supplementary Fig. 10D) are characterized by a relatively low UMIs/gene and, therefore, cells belonging to these two clusters of IRT003 were not included in the integrated dataset.

Since mouse samples were not FACS-sorted, additional filtering aiming to remove non-tumoral cells was performed based on the expression of Ptprc, Epcam, Smarcb1 genes (Supplementary Fig. 13). The three samples were first integrated using the CCA-based Seurat version 3.2.2 methods before UMAP embedding.

#### scRNA-seq data integration

scRNA-seq data integration was performed using the CCA-based approach described in Stuart et al.^86^ and implemented in Seurat version 3. Data integration quality was assessed by plotting the cell cycle phases on the integrated data (Supplementary Figs. 11D and 11D).

#### scRNA-seq cell clustering

PCA was applied to reduce the dimensionality of the integrated data using the *RunPCA()* function. The integrated data matrix was previously scaled using the *ScaleData()* function before PCA. The clustering was conducted using the graph-based modularity optimization Louvain algorithm implemented in Seurat v3. KNN graph is first built using the *FindNeighbors()* function on a user defined number of PCs. Then, the clustering was performed using the *FindClusters()* function with a specific resolution. Since the clustering result depends on the chosen number of PCs and the resolution value and we do not have an a priori about the expected number of clusters in our dataset, we choose to explore our data by running the clustering algorithm using different combination of number of PCs (from 12 to 50 increment by 1) and resolutions (from 0 to 1.5 increment by 0.1). The IKAP approach published in Chen et al.^87^ was used to perform this recursive clustering and to assess their stability. In addition, iterative clustering was also applied by removing cells belonging to well-defined clusters and re-running the clustering with the remaining cells. This approach leads to the identification of 10 clusters which can be obtained using a 12 PCs with a clustering resolution equals to 0.2 and recapitulating all the possible clusters identified throughout the analyses performed above. UMAP was used to visualize the clustering result in reduced two dimensions.

#### scRNA-seq cluster marker genes and cell type annotation

To biologically annotate the identified clusters, differential expression analyses in one versus others manner using the *FindAllMarkers()* function were carried out to identify marker genes for each cluster. The default Wilcoxon Rank Sum test was applied. Genes with a log_2_(fold-change) higher than 0.5, an adjusted *p*-value lower than 0.01 and detected in more than 25% of the cells of the given cluster were defined as gene markers for this cluster. These markers were investigated by knowledge-based using literature curations to identify the biological closest cell type of the cluster. In addition, on-line data bases such as http://mousebrain.org/, http://dropviz.org/, https://portal.brain-map.org/ and https://www.proteinatlas.org/ were used to explore marker genes cell type.

#### scRNA-seq gene regulatory network analysis

Gene regulatory network (GRN) analysis was conducted using the SCENIC framework^16^. For more details about the analysis steps, please refer to Sande et al.^88^.

The *pyscenic* programm version 0.10.3 was used to conduct the analysis. For the analysis of human samples, the TFs list (containing 1,839 human TFs) downloaded from https://github.com/aertslab/pySCENIC/blob/master/resources/ on August 7th, 2020, the cisTarget database (https://resources.aertslab.org/cistarget/) and the human motif annotation table downloaded from https://ressources.aertslab.org/cistarget/motif2tf/ on August 12th 2020 were for the analysist. For the analysis of mouse samples, the TFs list (containing 1721 mouse TF) downloaded from https://github.com/aertslab/pySCENIC/blob/master/resources/ on January 20th 2022, the cisTarget database and the mouse motif annotation table downloaded from https://ressources.aertslab.org/cistarget/motif2tf/ on January 20th 2022 were used. The regulon specificity score (RSS) was computed for each cluster and for each regulon in order to identify the most specific regulon compared to other clusters. Python version 3.6.11 was used for the analysis.

#### scRNA-seq trajectory inference (TI) and pseudotime analysis

TI analyses were conducted using reverse graph embedding approaches implemented in (1) ElPiGraph version 1.0.0 (Albergante et al., 2020) R package and based on the elastic principal graph^89^ and (2) the Monocle3 version 0.2.3.0 (Cao et al.^17^) R package and based on the Partition-based graph abstraction (PAGA)^90^. For the ElPiGraph tool, both the *computeElasticPrincipalTree()* and the *computeElasticPrincipalCurve()* function were run using 30 nodes. Lambda and Mu parameters were set to 0.01 and 0.1 respectively. All other parameters are set to their default values. For the Monocle3 tool, the dimensionality of the data was reduced using UMAP (based on 12 PCs) before cell clustering and graph inference. TI analyses were conducted within R version 4.0.2 environment.

#### scRNA-seq RNA velocity analysis

RNA velocity analysis was performed using the scVelo version 0.2.3 (Bergen et al.^19^) tool using the dynamical modeling. Spliced and unspliced matrices were generated separately for each sample in loom file using the velocito tool version 0.17.17^91^. RNA velocity was estimated using the marker genes for considered clusters and visualized on the embedding UMAP coordinates generated by Monocle3 (see TI analysis part). RNA velocity analysis was performed inside python version 3.7.8 environment.

#### scRNA-seq ligand-receptor interaction analysis

Ligand-receptor interaction analysis was performed using the cellPhoneDB framework^49^. To conduct this ligand-receptor interaction analysis, the *cellphonedb* binary (version 2.1.7) was run on the integrated expression matrix including all genes. Only ligand-receptor interaction with mean higher than 0.075 and a *p*-value lower than 0.05 were considered as significant interaction. The analysis was conducted inside Python 3.7.10 environment.

#### scRNA-seq differentiation state prediction

Cell differentiation state was estimated using the CytoTRACE^44^ tool (version 0.3.3, Gulati et al.^44^) with default options. CytoTRACE assesses the relative differentiation state of each cell using a predictive model based on the gene counts (number of expressed genes) and the average expression of genes that are highly correlated with gene counts (see Gulati et al.^44^ for more details). For each cell, it generates a score between 0 and 1 indicating a relatively more differentiated and less differentiated state. The analyses were conducted inside R 4.0.2 environment.

### Single nucleus data analysis

Single nucleus data of an ATRT sample (ATRT5) localized in the pineal area published in Jessa et al.^6^ was re-analyzed. Gene read counts per cell (generated by cellranger) were provided by the author. Very basic data filtering was applied. Nuclei having number of detected genes lower than the 5th percentile were filtered out. Sequencing depth normalisation was performed using the logNormalize function of Seurat (Version 3.2.2). Dimensionality reduction using PCA was applied to the dataset using the 2000 highly variable genes (Seurat flavor). Gene marker expressions were assessed using the heatmap density produced by the nebulosa^92^ R package (Version 1.0.1) on a UMAP embedding visualization based on the 12 first PCs.

### Reporting summary

Further information on research design is available in the Nature Portfolio Reporting Summary linked to this article.

### Supplementary information

Supplementary Information

Description of Additional Supplementary Files

Supplementary Data 1

Supplementary Data 2

Supplementary Data 3

Supplementary Data 4

Supplementary Data 5

Supplementary Data 6

Supplementary Data 7

Supplementary Data 8

Supplementary Data 9

Supplementary Data 10

Supplementary Data 11

Reporting Summary

### Source data

Source Data

## Data Availability

Raw and processed data included in this study have been deposited in NCBI’s Gene Expression Omnibus and accessible through GEO SuperSeries accession number GSE242090. Cell lines bulk RNA-seq data are available through GEO accession number GSE241733. Bulk RNA-seq data of mouse rhabdoid tumor model are accessible through GEO accession number GSE241734. Mouse gene expression array (Affymetrix®) from Han et al., 2016 are available in GEO under accession number GSE64019. Single cell RNA-seq data from mouse model are deposited in GEO under accession code GSE241736 Single cell RNA-seq data from human primary ATRT are deposited in GEO under accession code GSE241737 Bulk RNA-seq data of human primary ATRT are accessible through GSE241831 accession code. Bulk RNA-seq data from Andrianteranagna et al., 2021 are stored in GEO under accession code GSE175891. Bulk RNA-seq data from Leruste et al., 2019 are deposited in dbGaP database under accession code phs001915.v1.p1. DNA methylation array (Illumina Infinium MethylationEPIC) data are accessible through GSE242089 code. DNA methylation array data from Andrianteranagna et al., 2021 are available in GEO under accession number GSE175892. Source data are provided with the manuscript, without restriction. Source data are provided with this paper.

## Code availability

The codes are available in GitHub following the link: https://github.com/bourdeautlab/LobonIglesias2023.
