# Supplementary material for: Imaging and multi-omics datasets converge to define different neural progenitor origins for ATRT-SHH subgroups
Source: Nat Commun. 2023 Oct 20;14:6669. doi: 10.1038/s41467-023-42371-7 (PMC10589300; doi:10.1038/s41467-023-42371-7)
Supplement: Supplementary file 1 — Supplementary Information [file 41467_2023_42371_MOESM1_ESM.pdf]

Supplementary Fig. 1

A

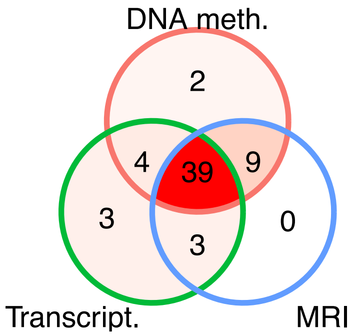

B

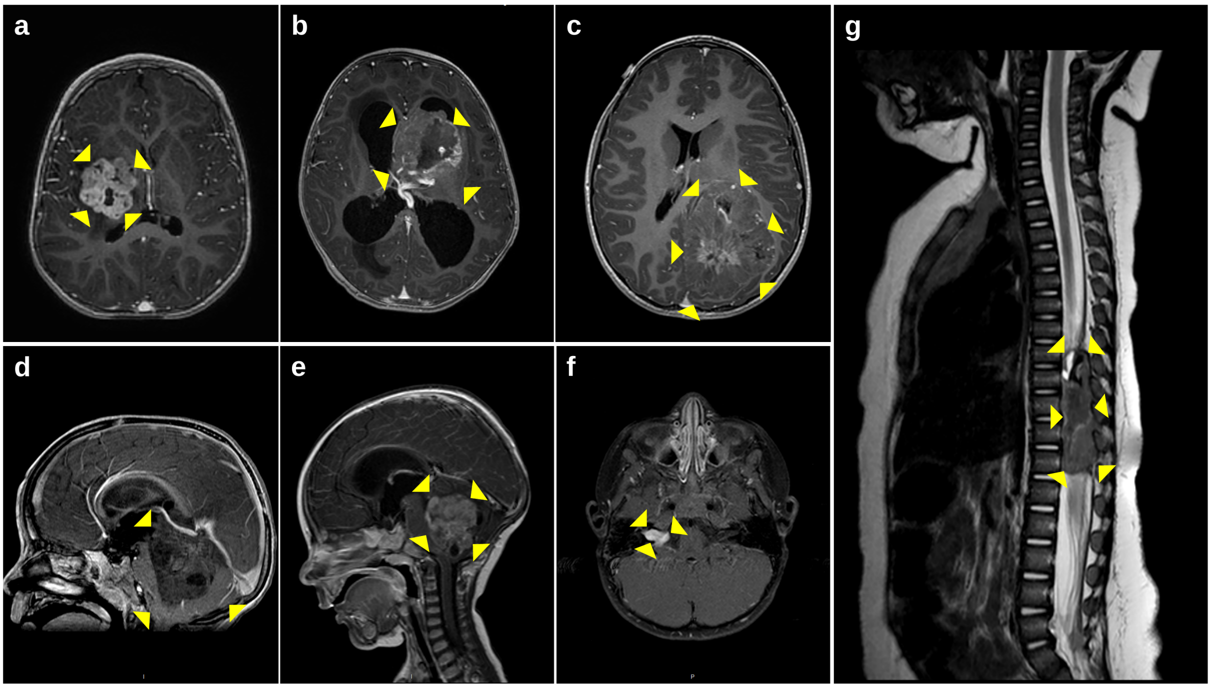

C

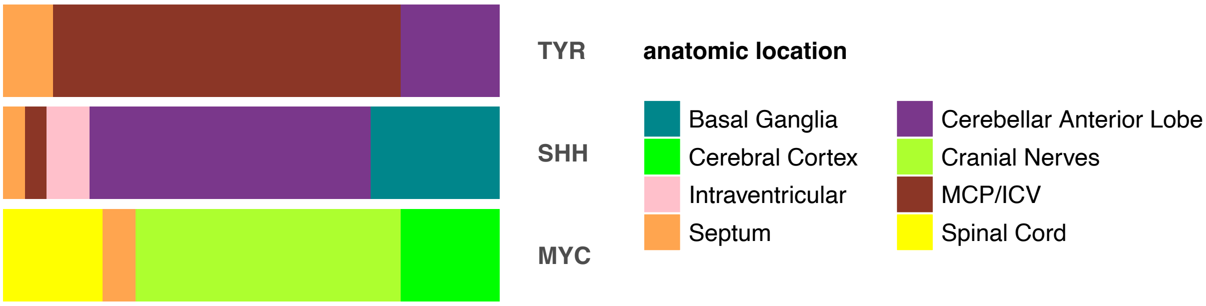

Supplementary Fig. 1. Radiological description of ATRTs' epicenter suggests clearly distinct origins for each molecular subtype.

(A) Venn diagram recapitulating the number of samples in each dataset: DNA methylation (DNA meth., red), magnetic resonance imaging (MRI, blue) and transcriptomic dataset (Transcrip.,green). DNA methylation data are obtained using the Illumina Human Infinium EPIC array and the transcriptomic data are from RNA sequencing (RNA-seq) technology.
(B) MR imaging localization of ATRT subtypes on T1-weighted images after injection of gadolinium chelate, on axial (a,b,c,f) or sagittal (d,e) plane (except g: sagittal T2-weighted image). (a) ATRT centered on the right basal ganglia (between the putamen and the thalamus). (b) ATRT in the frontal horn of the left lateral ventricle. (c) ATRT cortically located moving the adjacent parenchyme towards the ventricle. (d) ATRT centered in the cerebellar anterior lobe / quadrigeminal cistern area. (e) ATRT developed within the 4th ventricle from the inferior vermis. (f) ATRT in the right internal auditory canal, probably from 7th/8th cranial nerve. (g) ATRT located in the epidural space within the dorsal vertebral canal.
(C) Barplot showing the proportion of each tumor anatomical location for each ATRT molecular subgroup (TYR, SHH and MYC). These subgroups are defined according to DNA methylation dataset and the DKFZ Brain Tumor Classifier.

Supplementary Fig. 2

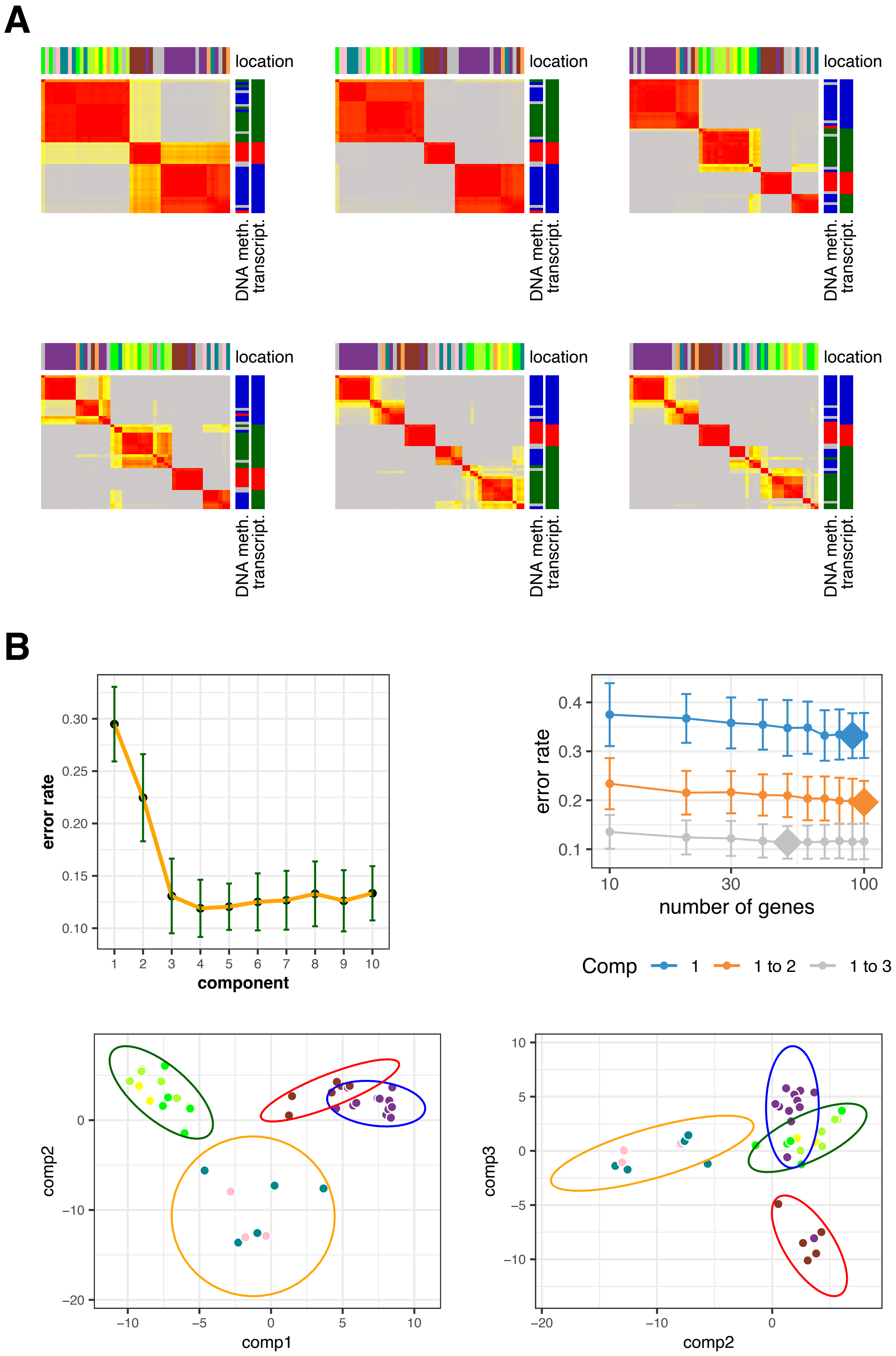

Supplementary Fig. 2. Integrative analysis identified four anatomical-molecular subgroups and splits SHH ATRT in two subgroups with distinct anatomical locations and transcriptional profiles.

(A) Consensus clustering of ATRT samples based on RNA-seq data (n=49 biologically independent samples) showing results from 2 to 7 classes. Top annotation indicates anatomical location. NA: unknown anatomical location. Right annotation indicates molecular subgroups according respectively to transcriptomics (RNA-seq, see Figure 2a) and DNA methylation profile (EPIC array, DKFZ brain tumor classifier v11b4, see Figure 1e).

(B) sPLS-DA analysis (n=39 biologically independent samples): top left panel: classification error rate (Y axis) for each number of PCs (X axis, comp.); top right: error rate (X axis) for each number of genes (Y axis) considered for each of the 3 first components (comp. 1: blue, comp. 2: orange, comp. 3: grey); bottom left: individual plot with comp. 1 and comp. 2; bottom right: individual plot with comp. 2 and comp. 3.

Supplementary Fig. 3

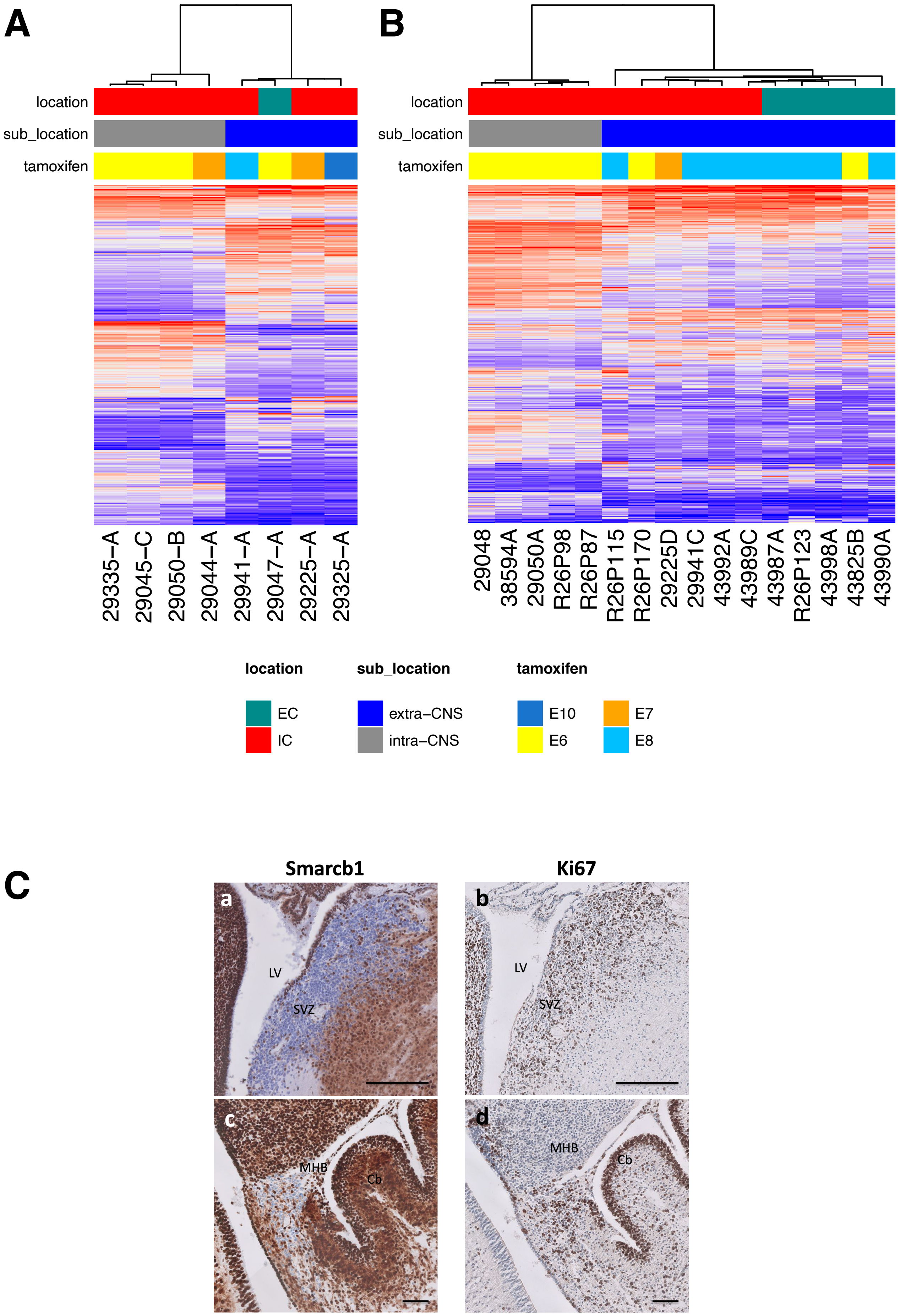

Supplementary Fig. 3. Mouse RT clustered in 2 transcriptomics subgroups corresponding to anatomical localization and *Smarcb1* inactivation time.

Unsupervised hierarchical clustering of mouse RT based on transcriptomic dataset:

(A) RNA-seq (n=16) and (B) gene expression microarray (n=8, reanalysis from Han *et al.*, 2016). For both datasets, the hierarchical clustering was performed using the 1 - Pearson coefficient as distance metric and Ward method as linkage. Only the 2000 most variable genes (based on IQR value of normalized count/intensity) were considered, and they were clustered using the Euclidean distance metric and the Ward linkage.

(C) BAF47 (a, c) and Ki67 (b, d) immunostaining in the vicinity of the lateral ventricle (LV) and the subventricular zone (SVZ) (a, b) and in the region of the midbrain-hindbrain boundary (MHB) and upper cerebellum (Cb) (c, d). BAF47 negative staining tumor (or cells) are positive for Ki67 staining. Scale bar: 100 µm.

Supplementary Fig. 4

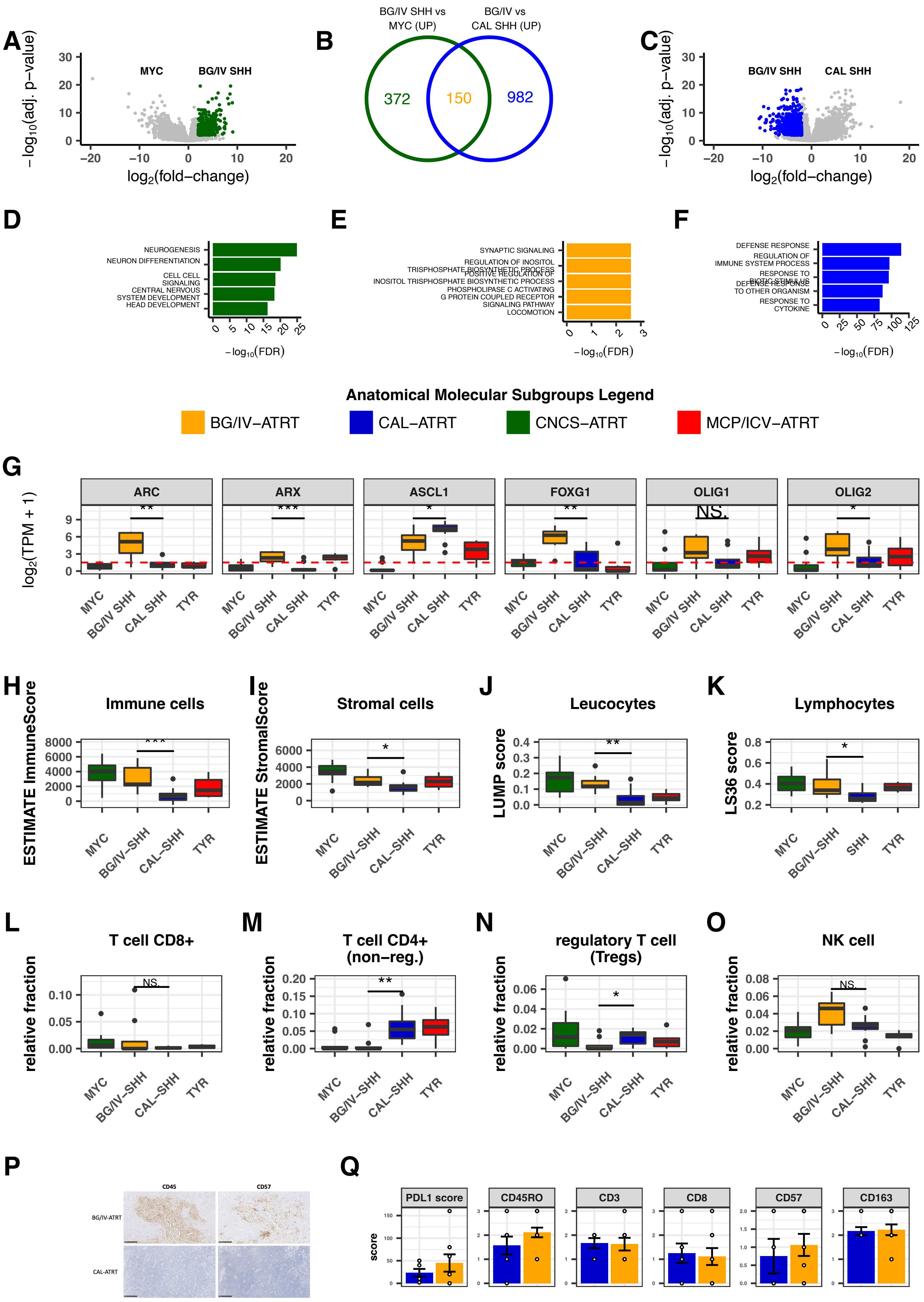

Supplementary Fig. 4. BG/IV-SHH ATRT tumors are characterized by both neuronal signatures and immune infiltration.

(A) Differential gene expression analyses between BG/IV-SHH ATRT and CNCS-MYC ATRT and (C) between BG/IV-SHH ATRT and CAL-SHH ATRT. Genes having log2 fold-change higher than 2 and an adjusted p-value lower than 0.001 are considered as significantly differentially expressed (DEG). Negative binomial GLM and Wald test were applied for gene expression comparison and generated p-values were corrected using the Benjamini and Hochberg method.

(B) Venn diagram showing the overlap between the DEG overexpressed in BG/IV-SHH shown in green (A) and blue (B). 372 genes are overexpressed in BG/IV-SHH only when compared with CNCS-MYC; 982 genes are overexpressed in BG/IV-SHH only when compared with CAL-SHH; 150 genes are overexpressed in BG/IV-SHH in the two comparisons. (D, E, F) Gene ontology analyses performed on the 372 (D), 150 (E) and 982 (F) genes shown in the venn diagramm.

(G-O) Boxplots of gene expression and immune cell content estimations for which the boxes represent the interquartile ranges while the whisker bonds indicate the highest and smallest values within 1.5 times interquartile range above and below the 75th and 25th quantiles, respectively. Wilcoxon signed-rank test were applied to compare mean gene expression levels, immune scores and cell type relative contents between BG/IV-SHH and CAL-SHH anatomical molecular subgroups (*: p-value < 0.05; **: p-value < 0.01; *: p-value < 0.001)

(G) Boxplot of BG/IV-SHH specific gene expression across the 4 anatomico-molecular subgroups (n=39 biologically independent samples: $n_{CNCS\text{-}MYC}$=13, $_{nBG/IV\text{-}SHH}$=8, $n_{CAL\text{-}SHH}$ =12, $n_{MCP/ICV\text{-}TYR}$ = 6); comparison of gene expression levels between BG/IV-SHH and CAL-SHH Wilcoxon t-test p-values: ARC=0.015, ARX=0.00048, ASCL1=0.031, FOXG1= 0.0011, OLIG1=0.069, OLIG2=0.047.

(H,I) Results obtained with ESTIMATE algorithm, based on transcriptomic data, showing the immune infiltration (H) and stromal score (I) for each subgroup (n=39 biologically independent samples: $n_{CNCS\text{-}MYC}$=13, $_{nBG/IV\text{-}SHH}$=8, $n_{CAL\text{-}SHH}$ =12, $n_{MCP/ICV\text{-}TYR}$ = 6). Comparison of BG/IV-SHH versus CAL-SHH immune and stromal scores result in p-value of 0.00071 and 0.016 respectively.

(J,K) Results obtained with LUMP and LS36 scoring, based on methylation dataset showing the leucocytes (J) and the lymphocytes (K) infiltration for each subgroup (n=42 biologically independent samples: $n_{CNCS\text{-}MYC}$=14, $n_{BG/IV\text{-}SHH}$=8, $n_{CAL\text{-}SHH}$ =12, $n_{MCP/ICV\text{-}TYR}$ =8). Comparison

of BG/IV-SHH versus CAL-SHH LUMP and LSl scores result in p-value of 0.0075 and 0.028 respectively.
(L-O) Results obtained with quanTIseq, a deconvolution approach based on transcriptomic dataset, showing the immune cells content in each subgroup (n=39 biologically independent samples: $n_{CNCS\text{-}MYC}$=13, $n_{BG/IV\text{-}SHH}$=8, $n_{CAL\text{-}SHH}$ =12, $n_{MCP/ICV\text{-}TYR}$ = 6). Comparison of BG/IV-SHH versus CAL-SHH relative fraction of T cell CD8+, non-regulatory T cells CD4+, regulatory T cells and NK cell yield in p-value sof 0.47, 0.0026, 0.042, 0.00071 and 0.069 respectively.

(P) Immunostaining with anti CD45 anti CD57 antibodies on representative BG/IV-ATRT and CAL-ATRT samples.
(Q) Barplot of the immunohistochemistry score of a series immune cell markers on a collection of ATRTs. Color codes: blue for CAL-SHH (n=6) and orange for BG/IV-SHH (n=9). Error bars, represent the standard error of mean.

Supplementary Fig. 5

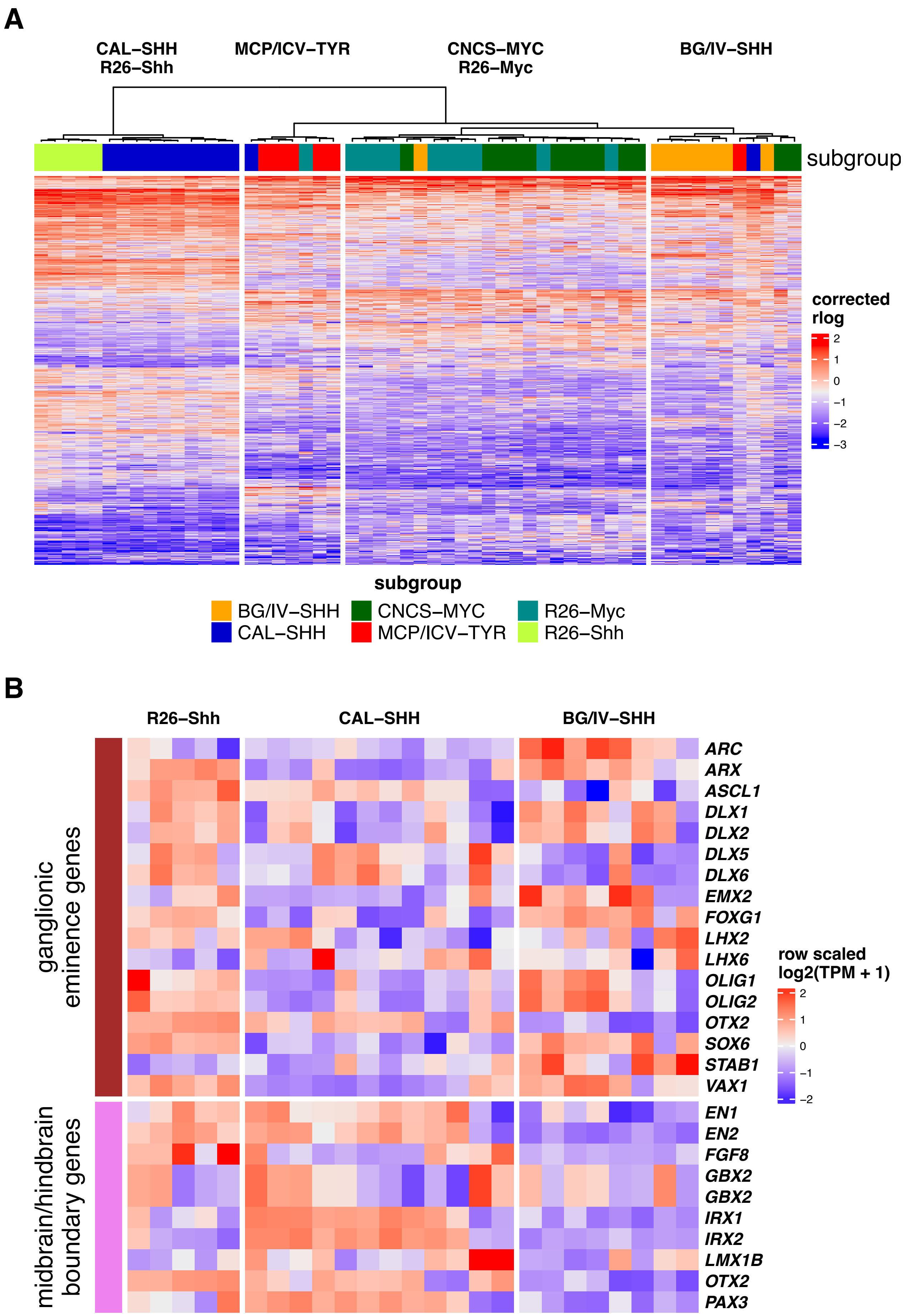

Supplementary Fig. 5. Integration of human and mouse ATRT transcriptomics profile

(A) Unsupervised hierarchical clustering of combined mouse and human samples. The two datasets were merged using the orthologous genes between the mouse and human. Organism effect were regressed out using linear model.

(B) Gene expression heatmap of ganglionic eminence genes and midbrain hindbrain genes in mouse R26-Shh, human BG/IV-SHH and human CAL-SHH samples.

Supplementary Fig. 6

A

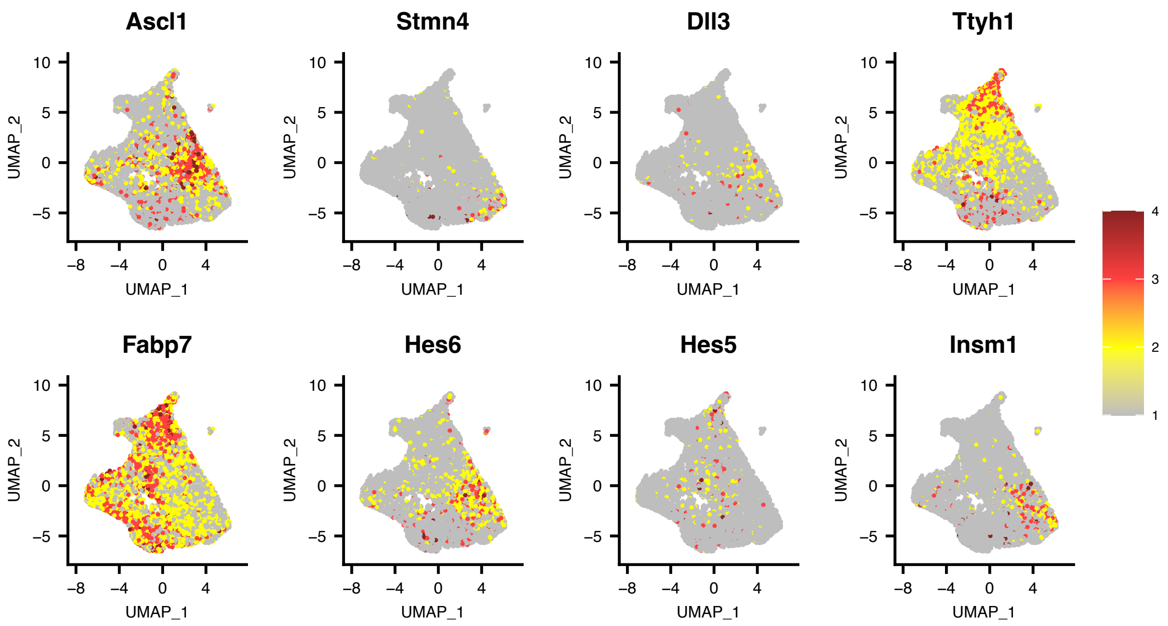

B

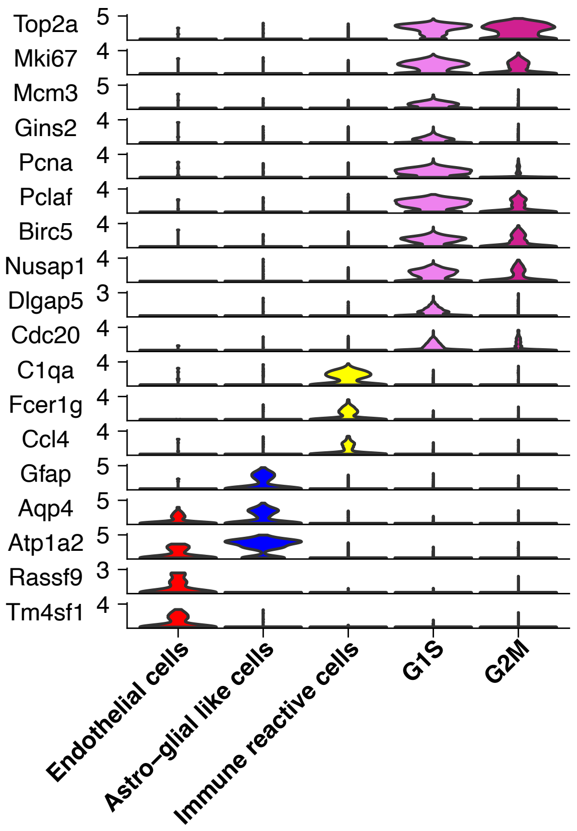

Supplementary Fig. 6. Transcriptional intratumoral heterogeneity of mouse ATRT Shh.

(A) Heatmap of ATRT-SHH gene signatures (Johann et al., 2016; Torchia et al., 2016) expression on the UMAP of integrated mouse RT *Shh* samples (n=3).

(B) Violin plot of marker genes of endothelial, immune reactive and cycling (G1S, G2M), cell populations.

Supplementary Fig. 7

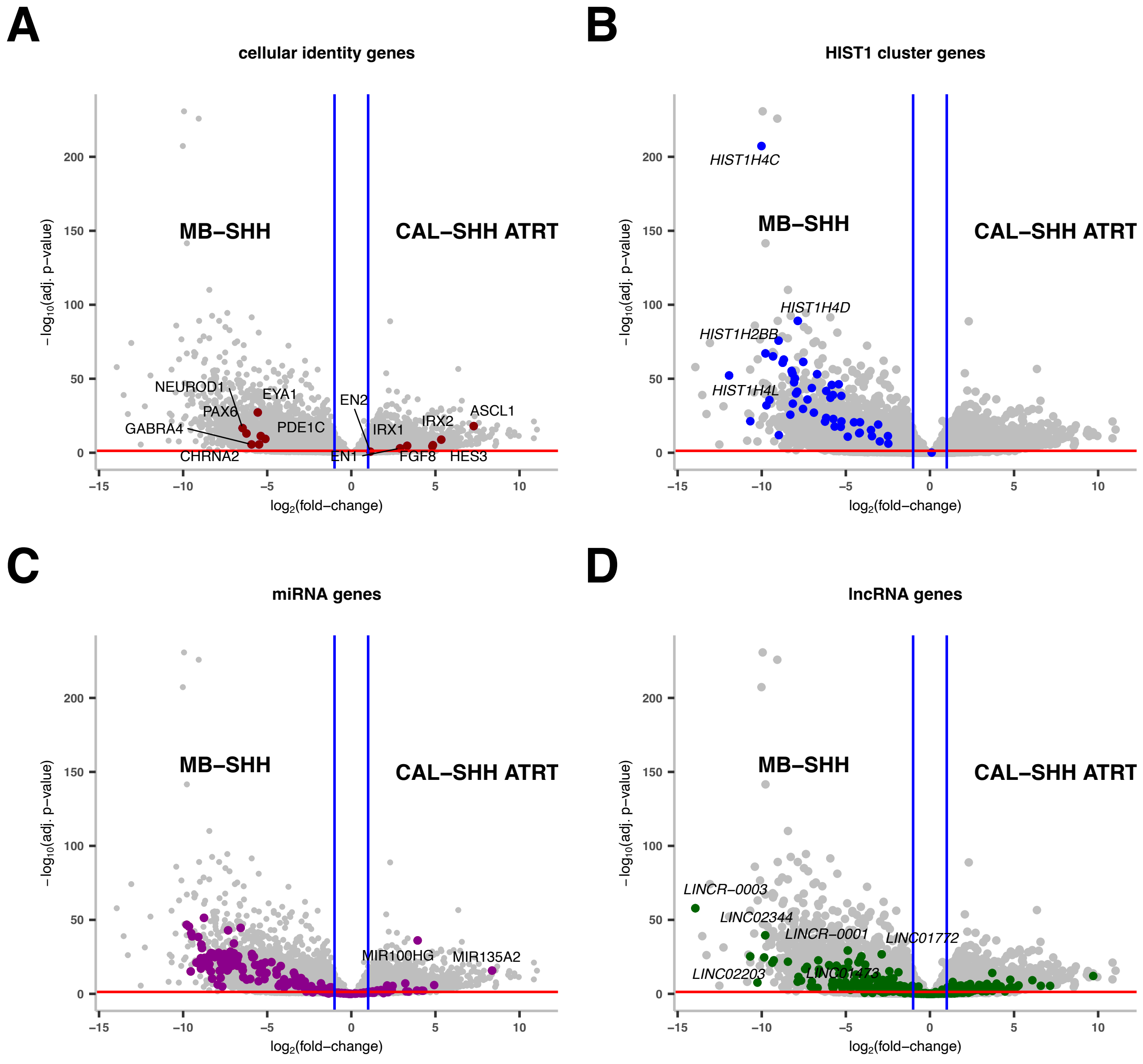

Supplementary Fig. 7. Differential expression analyses CAL-ATRT versus SHH-MB. Volcano plot showing the result of differential expression analysis between CAL-ATRT and SHH-MB: genes related to cellular identity (A), HIST1 genes (B), miRNA genes (C) and lncRNA genes (D) are highlighted and labelled. Negative binomial GLM and Wald test were applied for gene expression comparison and generated p-values were corrected using the Benjamini and Hochberg method.

Supplementary Fig. 8

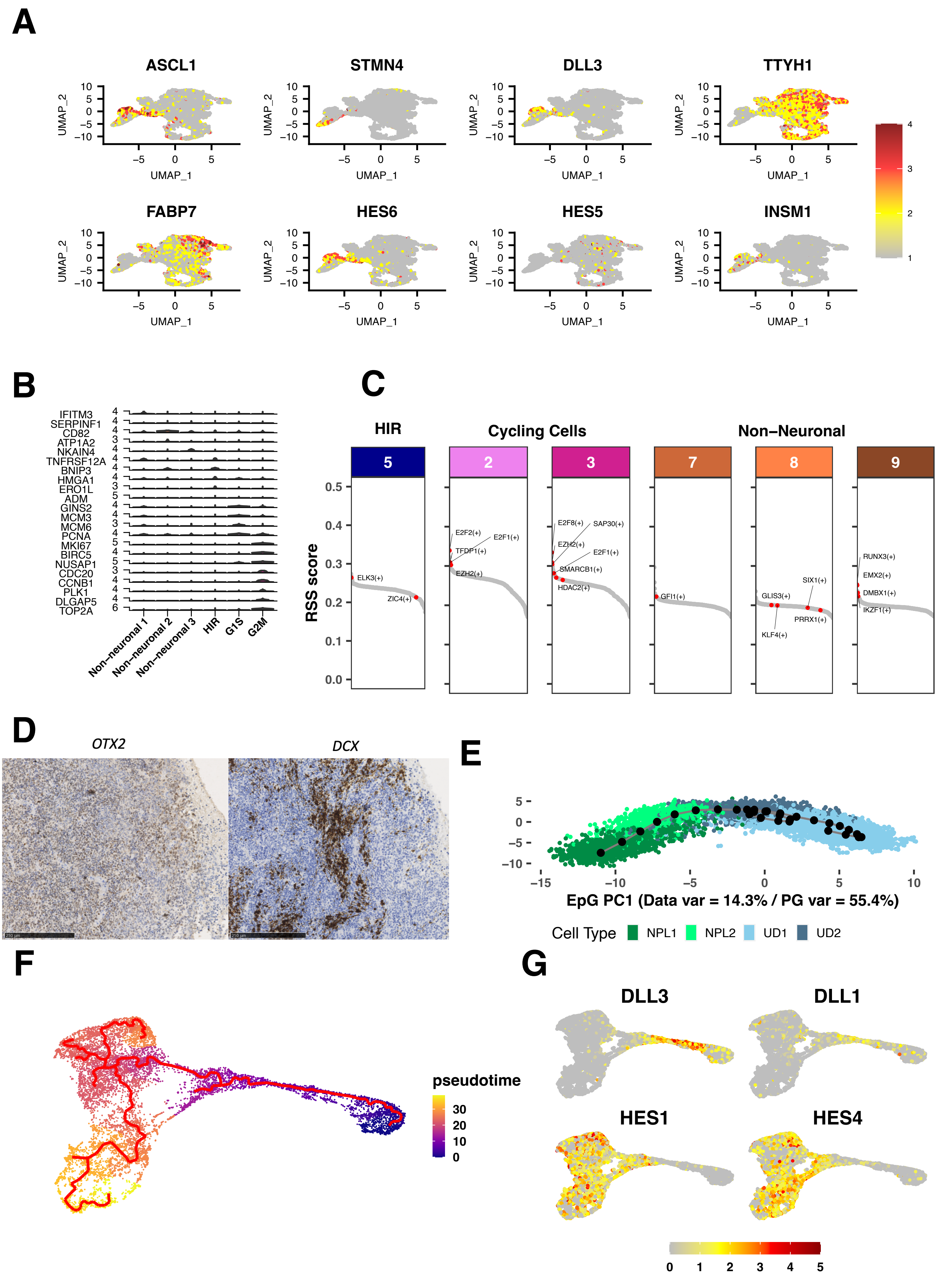

Supplementary Fig. 8. Transcriptional intratumoral heterogeneity and putative cells of origin of human CAL SHH ATRT.

(A) Heatmap of ATRT-SHH gene signatures (Johann et al., 2016; Torchia et al., 2016) expression on the UMAP of integrated human CAL-SHH ATRT samples (n=3).

(B) Violin plot of marker genes of cycling cells (G1S, G2M), HIR and non-neuronal cell populations.

(C) Regulon specificity score (RSS) for each cell cluster. Regulons (TF and their direct targets) are ordered along the X axis according to their RSS score (Y axis). Interesting regulons are highlighted in red and labelled.

(D) Immunohistochemistry staining using of OTX2 and DCX antibodies on a representative CAL-ATRT sample.

(E) Trajectory inference analysis using elPiGraph show a transition path from NPL1, NPL2 to undifferenciated cells. Dark green: NPL1 cells, light green: NPL2 cells, light blue: undifferentiated cells

(F) Pseudotime computed with Monocle3 after considering the NPL1 as the root of the trajectory

(G) Heatmap showing the expressin of NOTCH pathway genes using the UMAP embedding obtained from the trajectory analysis performed with Monocle3. The color gradient indicates the expression levels , from the lowest (grey) to the highest (red).

Supplementary Fig. 9

A

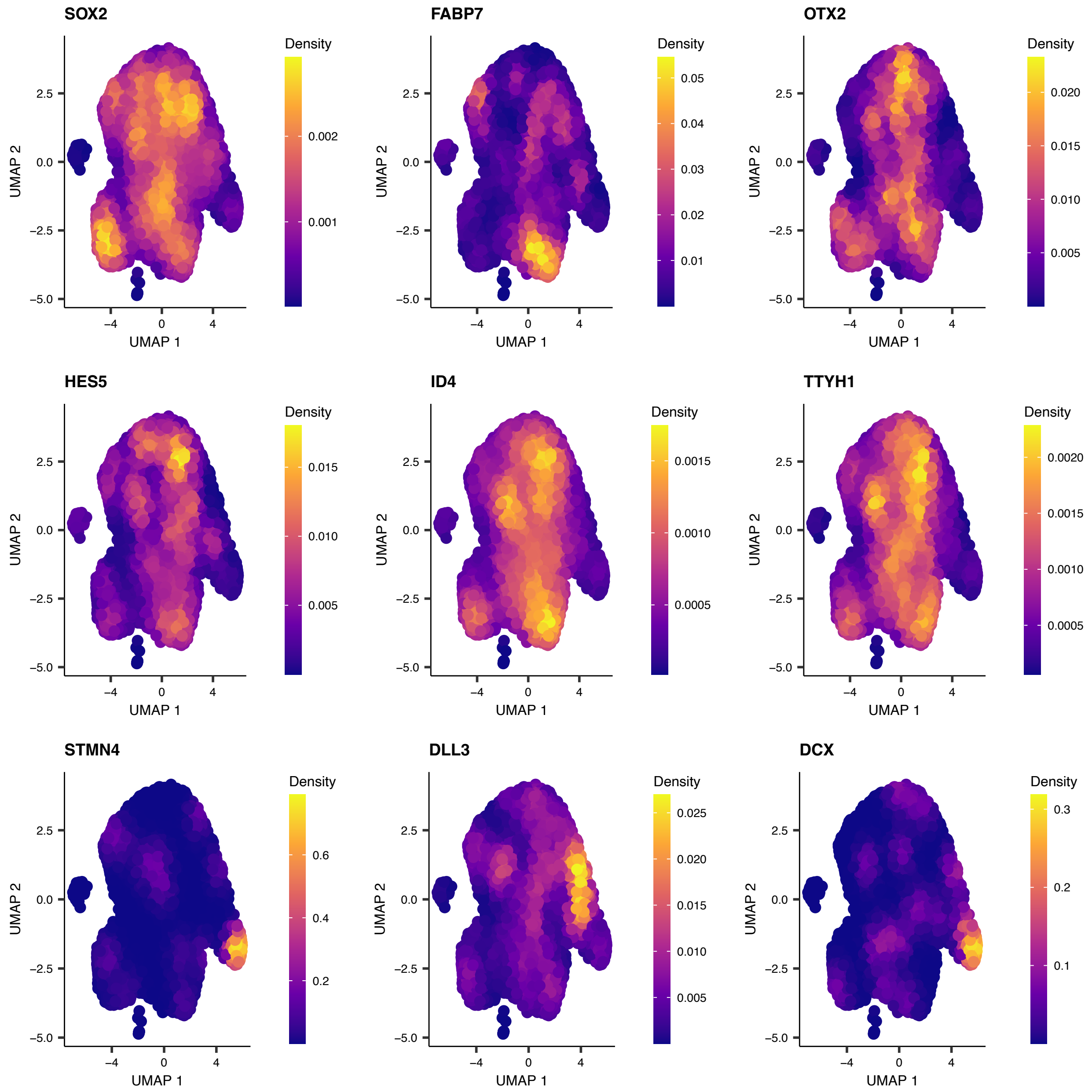

B

	CHLA-02	IC-032	
IC50	127.2	46.08	μM

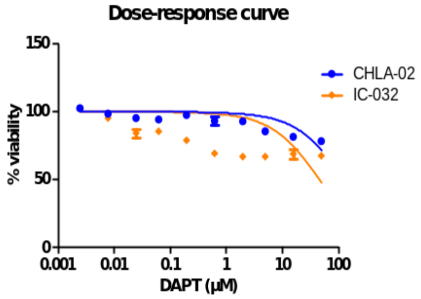

Supplementary Fig. 9. CAL-ATRT transcriptional intratumoral heterogeneity on previously published samples and response to gamma-secretase inhibitors in SHH ATRT cell lines

A. Gene expression density of committed (*STMN4, DLL3, DCX*) and less differentiated cell markers (*SOX2, FABP7, OTX2, HES5, ID4, TTYH1*) markers in ATRT5 sample (reanalysis from Jessa et al., 2019). B. Dose response curve assessed as percentage of cell viability of two ATRT cell lines, upon treatment with DAPT.

Supplementary Fig. 10

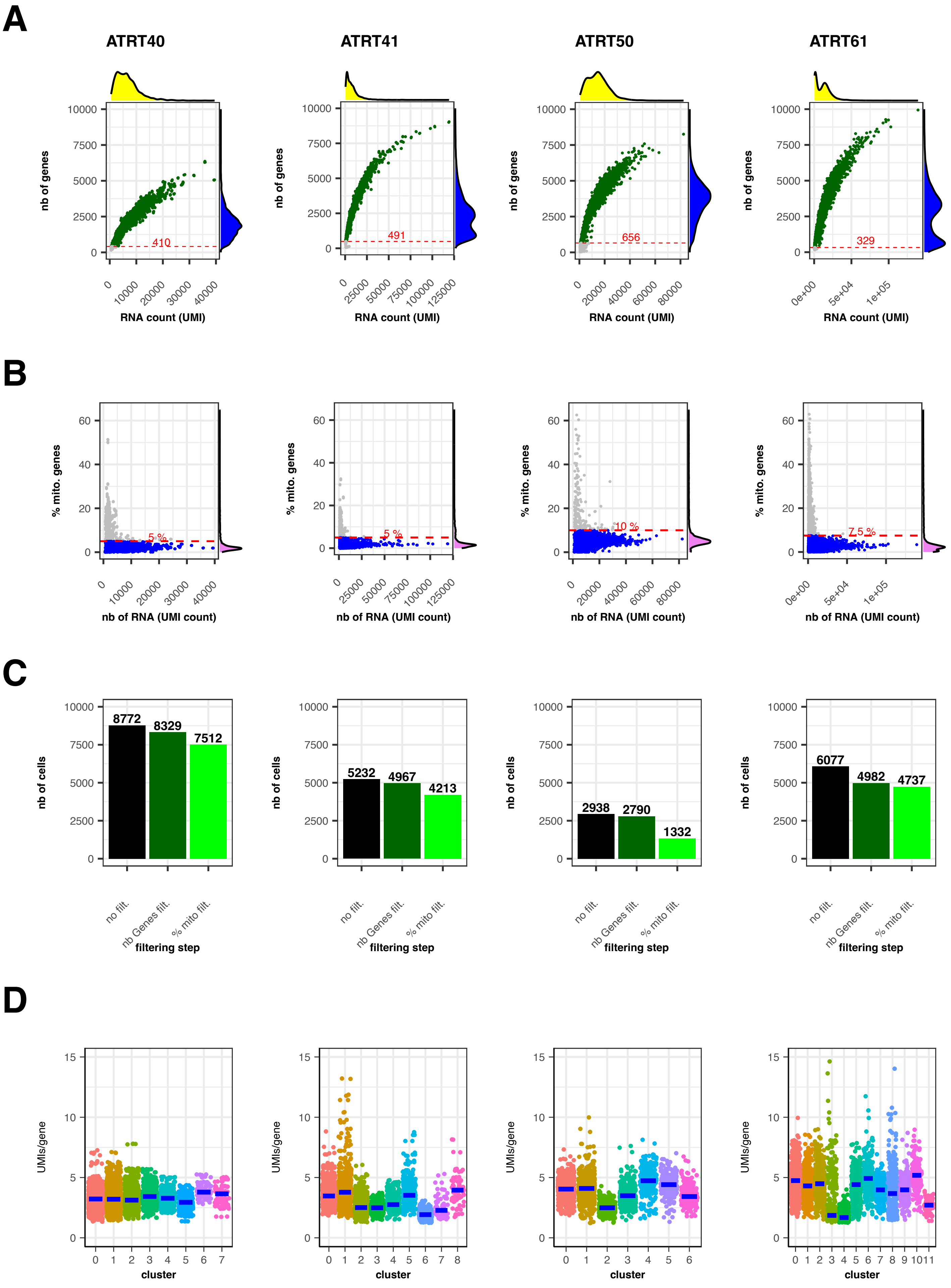

Supplementary Fig. 10. Human single cell filtering.

(A-C) Adaptive filtering based on the number of gene detected and the mitochondrial transcript content. Individually, for each sample, (A) cells having the number of genes lower than the 5th percentile value (indicated in red) are filtered out; (B) cells having a high content of mitochondrial transcript (above the indicated red dashed line) are filtered out; (C) Barplot showing, for each sample, the total number of cells before filtering (black), the number of cells after removing cells with low number of detected genes (dark green) and the number of cells after removing high content of mitochondrial transcript (light green).

(D) Cell clustering was performed with individual sample to identify cells that probably clustered due to technical variation. Therefore, for IRT003 sample, cells belonging to cluster 3 and 4 were filtered out before data integration because they show a relatively low number of UMI per gene.

Supplementary Fig. 11

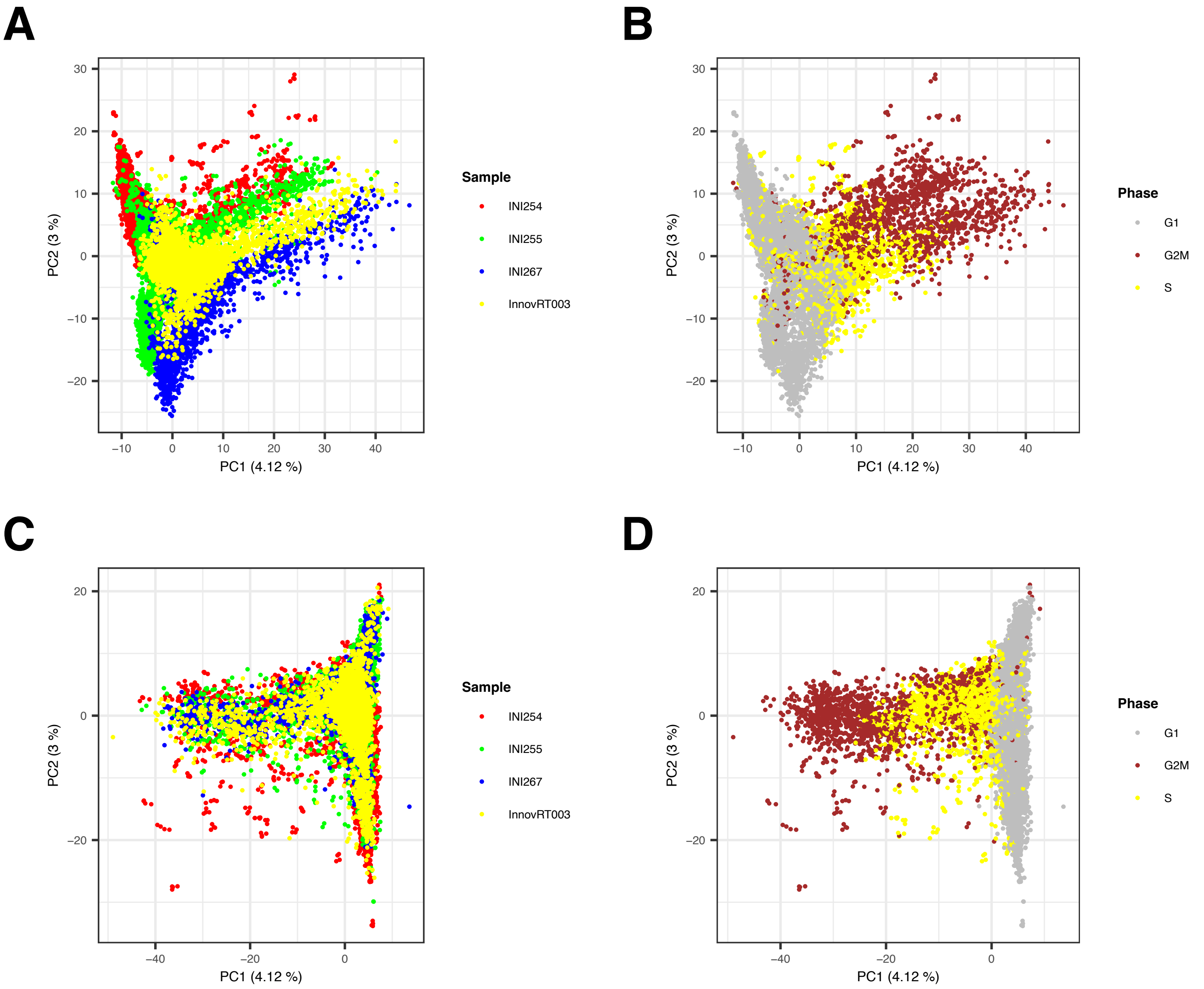

Supplementary Fig. 11. Human single cell data integration

Data integration using the CCA-based method implemented in Seurat version 3.

(A-B) PCA representation of the 4 samples before data integration where the sample origin (A) and the cell cycle phase (B) of each cell are indicated by different colors.

(C-D) PCA representation of the 4 samples after data integration where the sample origin (C) and the cell cycle phase (D) of each cell are indicated by different colors.

Supplementary Fig. 12

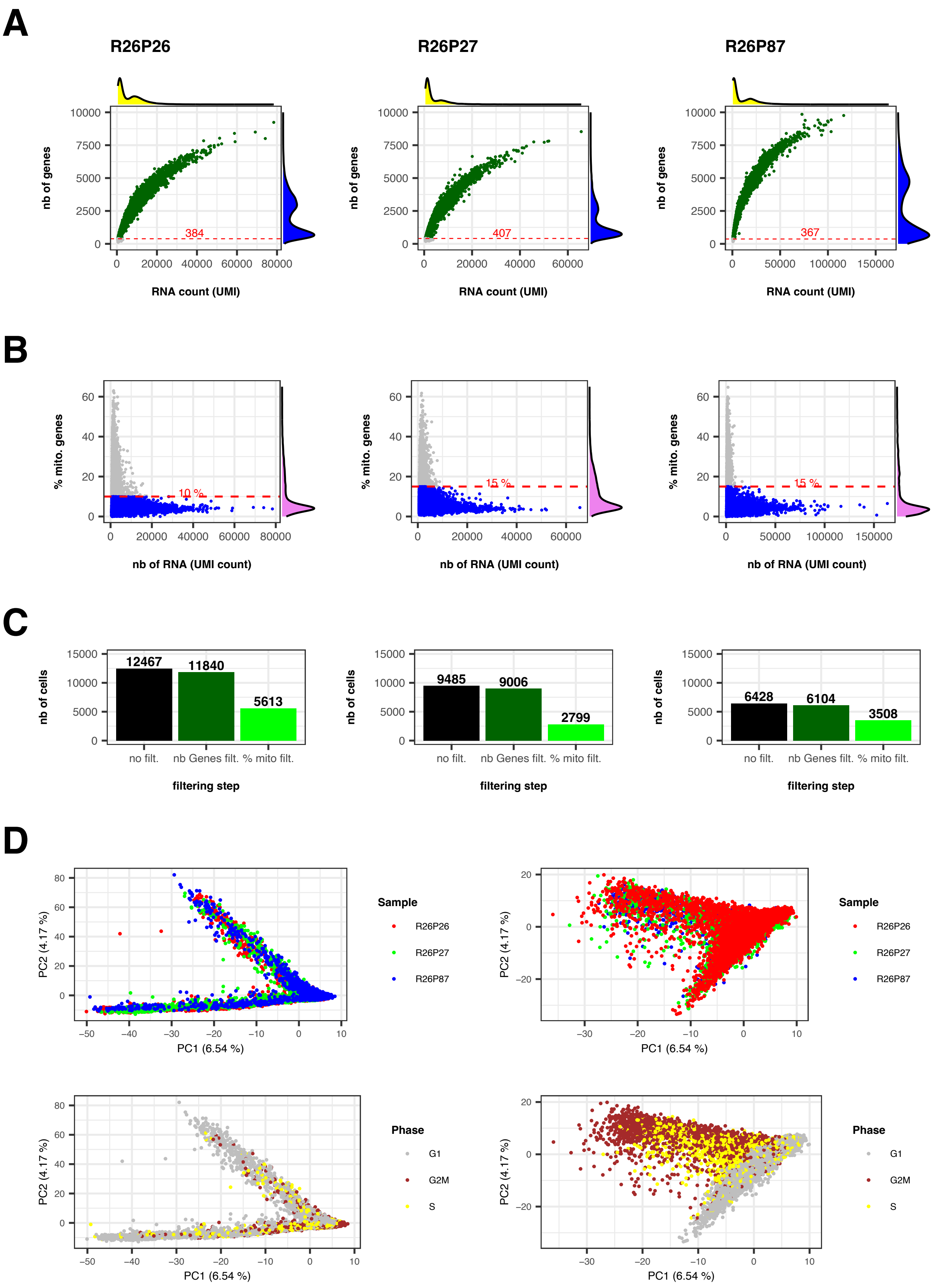

Supplementary Fig. 12. Mouse single cell filtering and data integration.

(A-C) Adaptive filtering based on the number of gene detected and the mitochondrial transcript content. For each sample, (A) cells having the number of genes lower than the 5th percentile value (indicated in red) are filtered out; (B) cells having a high content of mitochondrial transcript (above the indicated red dashed line) are filtered out; (C) Barplot showing, for each sample, the total number of cells before filtering (black), the number of cells after removing cells with low number of detected genes (dark green) and the number of cells after removing high content of mitochondrial transcript (light green).

(D) Data integration using the CCA-based method implemented in Seurat v3. PCA representation of the 3 samples before data integration where the sample origin (top left) and the cell cycle phase (bottom left) of each cell are indicated by different colors. PCA representation of the 3 samples after data integration where the sample origin (top right) and the cell cycle phase (bottom right) of each cell are indicated by different colors.

Supplementary Fig. 13

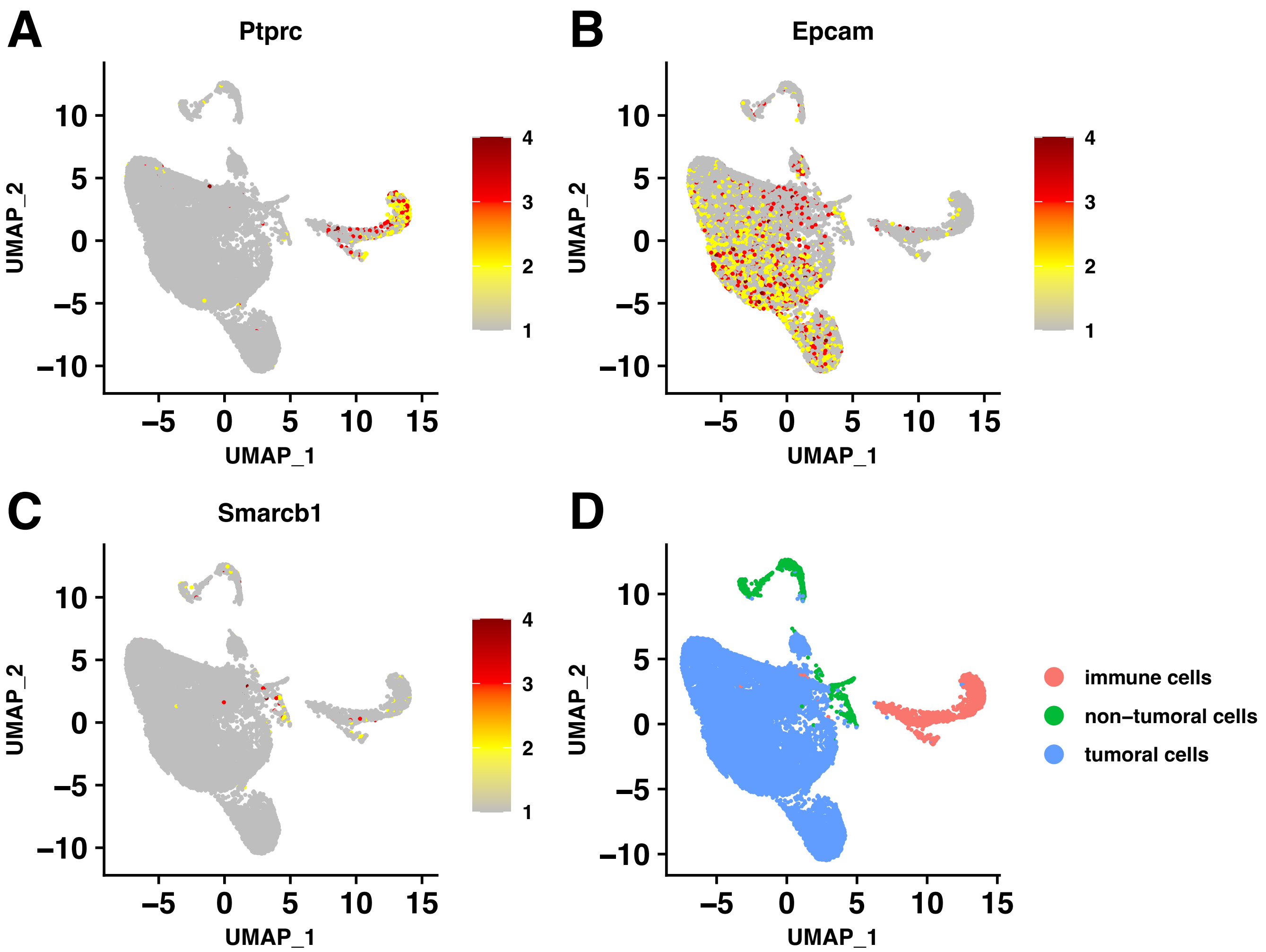

Supplementary Fig. 13. Mouse scRNA-seq non-tumoral cell filtering.

UMAP embedding of the integrated scRNA-seq data before removing non-tumoral cells showing: the expression of *Ptprc* gene, used as positive marker of immune cells (A), the expression of *Epcam* gene (B), used as positive marker of tumoral cells, the expression of *Smarcb1* gene (C), used as negative marker of rhabdoid tumor cells, all cells colored by their assigned identity (D).
