# Supplementary material for: Imaging and multi-omics datasets converge to define different neural progenitor origins for ATRT-SHH subgroups
Source: Nat Commun. 2023 Oct 20;14:6669. doi: 10.1038/s41467-023-42371-7 (PMC10589300; doi:10.1038/s41467-023-42371-7)
Supplement: Supplementary file 2 — Description of Additional Supplementary Files [file 41467_2023_42371_MOESM2_ESM.pdf]

Description of Additional Supplementary Files

SUPPLEMENTARY DATA LEGEND

Supplementary Data 1: Patient and sample information

Information corresponding to each of the 61 human ATRT samples: (1) minimal clinical features of the patients from whom the sample was collected ; (2) anatomical location for samples with available MRI ; (3) age at diagnosis ; (4) RNA-seq subgrouping for samples with available RNA-seq data, NA (Not Available) otherwise ; (5) DNA methylation subgroup for samples with availbable DNA methylation array, NA otherwise ; (6) DKFZ brain tumor ATRT subgrouping for samples with available DNA methylation array, NA otherwise ; (7) DKFZ brain tumor classifier score for samples with available DNA methylation array data ; (8) gene expression profile at the single cell level.

Supplementary Data 2: sPLS-DA gene contribution in the 3 first principal components

List of the genes that are contributing in the 3 first principal components of the Sparse Partial Least Squares Discriminant Analysis (sPLS-DA); the respective scores of contribution in each component. sPLS-DA were conducted using the *mixOmics* framework (version 6.14.1) Bioconductor R package. The optimal number of components as well as the number of genes per component were determined by running the *perf()* and *tune.splsda()* functions using 3-fold cross-validation repeated 50 times. Finally, sPLS-DA analysis was run using the *splsda()* function using 3 components with respectively the 90, 100 and 50 previously selected genes (supplementary Fig. 2B).

Supplementary Data 3: Mouse sample information

Information corresponding to each of the 62 mouse samples : (1) mouse features ; (2) Tamoxifen treatment date ; (3) age in days ; (4) sex, F: female, M: male, NA: not available ; (5) tumor location, IC: intracranial, EC: extracranial ; (6) tumor sub-location, CNS: Central Nervous System ; (7) RNAseq/Affy, gene expression profile analysed using RNA-seq or Affymetrix microarray ; (8) subgroup ; (9) scRNAseq, gene expression profile analysed at the single cell level.

Supplementary Data 4: List of differentially expressed genes for each anatomical-molecular subgroup in a « one versus all others » manner

List of the 100 most differentially expressed genes (based on fold-change of genes with a corrected *p*-value less than 0.05) between anatomical-molecular subgroups in a "one versus all others" manner ($n_{CNCS\text{-}MYC}$ = 13, $n_{BG/IV\text{-}SHH}$ = 8, $n_{CAL\text{-}SHH}$ =12, $n_{MCP/ICV\text{-}TYR}$ = 6). Differential gene expression analyses were performed using *DESeq()* function of the *DESeq2* Bioconductor R package (version 1.30.1). Size factor estimation, dispersion estimation, binomial negative GLM fitting and Wald test were applied on

filtered raw counts. Resulting *p*-values were corrected using the Benjamini and Hochberg method (a.k.a. FDR).

Supplementary Data 5: Differential gene expression analysis between BG/IV SHH and MYC

Result of the differential gene expression analyses between BG/IV SHH (n = 8) and MYC (n = 13) ATRT subtypes. Differential gene expression analyses were performed using *DESeq()* function of the *DESeq2* Bioconductor R package (version 1.30.1). Size factor estimation, dispersion estimation, binomial negative GLM fitting and Wald test were applied on filtered raw counts. Resulting *p*-values were corrected using the Benjamini and Hochberg method (a.k.a. FDR).

Supplementary Data 6: Differential gene expression analysis between BG/IV SHH and CAL SHH

Result of the differential gene expression analyses between BG/IV SHH (n = 8) and CAL SHH (n = 12) ATRT subtypes. Differential gene expression analyses were performed using *DESeq()* function of the *DESeq2* Bioconductor R package (version 1.30.1). Size factor estimation, dispersion estimation, binomial negative GLM fitting and Wald test were applied on filtered raw counts. Resulting p-values were corrected using the Benjamini and Hochberg method (a.k.a. FDR).

Supplementary Data 7: Gene markers of the 13 mouse R26 single cell clusters

List of gene markers of each of the 13 cell clusters identified in the single cell data of integrated mouse R26-Shh samples (n = 3). Cluster gene markers were identified by differential expression analyses in « one versus others » manner using the *FindAllMarkers()* function of the Seurat R package (version 3.2.2). The default Wilcoxon Rank Sum test was applied. Genes with a log2(fold-change) higher than 0.5, an adjusted *p*-value lower than 0.01 and detected in more than 25 % of the cells of the given cluster were considered as gene markers for this cluster.

Supplementary Data 8: Differential gene expression analysis between CAL SHH and MB SHH

Result of the differential gene expression analyses between CAL SHH ATRT subtype (n = 12) and SHH medulloblastoma subtype (n = 7). Differential gene expression analyses were performed using *DESeq()* function of the *DESeq2* Bioconductor R package (version 1.30.1). Size factor estimation, dispersion estimation, binomial negative GLM fitting and Wald test were applied on filtered raw counts. Resulting *p*-values were corrected using the Benjamini and Hochberg method (a.k.a. FDR).

Supplementary Data 9: Gene markers of the 10 human ATRT CAL SHH single cell clusters

List of gene markers of each of the 10 cell clusters identified in the single cell data of integrated ATRT CAL SHH samples (n = 4). Cluster gene markers were identified by differential expression analyses in « one versus others » manner using the *FindAllMarkers()* function of the Seurat R package (version

3.2.2). The default Wilcoxon Rank Sum test was applied. Genes with a log2(fold-change) higher than 0.5, an adjusted *p*-value lower than 0.01 and detected in more than 25 % of the cells of the given cluster were considered as gene markers for this cluster.

Supplementary Data 10: Differential gene expression analysis between DAPT and DMSO treated CHLA-02 cell line

Result of the differential gene expression analyses between DAPT (n=3) and DMSO (n=3) treated CHLA-02 cell lines. The analysis was performed using *DESeq()* function of the *DESeq2* Bioconductor R package (version 1.30.1). Size factor estimation, dispersion estimation, binomial negative GLM fitting and Wald test were applied on filtered raw counts. Resulting p-values were corrected using the Benjamini and Hochberg method (a.k.a. FDR).

Supplementary Data 11: Differential gene expression analysis between DAPT and DMSO treated IC-032 cell line

Result of the differential gene expression analyses between DAPT (n=3) and DMSO (n=3) treated IC-032 cell line. The analysis was performed using *DESeq()* function of the *DESeq2* Bioconductor R package (version 1.30.1). Size factor estimation, dispersion estimation, binomial negative GLM fitting and Wald test were applied on filtered raw counts. Resulting p-values were corrected using the Benjamini and Hochberg method (a.k.a. FDR).
