# Supplementary material for: Imaging and multi-omics datasets converge to define different neural progenitor origins for ATRT-SHH subgroups
Source: Nat Commun. 2023 Oct 20;14:6669. doi: 10.1038/s41467-023-42371-7 (PMC10589300; doi:10.1038/s41467-023-42371-7)
Supplement: Supplementary file 14 — Reporting Summary [file 41467_2023_42371_MOESM14_ESM.pdf]

nature portfolio

Corresponding author(s): Franck Bourdeaut

Last updated by author(s): 2023/09/25

# Reporting Summary

Nature Portfolio wishes to improve the reproducibility of the work that we publish. This form provides structure for consistency and transparency in reporting. For further information on Nature Portfolio policies, see our Editorial Policies and the Editorial Policy Checklist.

## Statistics

For all statistical analyses, confirm that the following items are present in the figure legend, table legend, main text, or Methods section.

| n/a | Confirmed | |
|---|---|---|
| ☐ | ☒ | The exact sample size (*n*) for each experimental group/condition, given as a discrete number and unit of measurement |
| ☐ | ☒ | A statement on whether measurements were taken from distinct samples or whether the same sample was measured repeatedly |
| ☐ | ☒ | The statistical test(s) used AND whether they are one- or two-sided<br>*Only common tests should be described solely by name; describe more complex techniques in the Methods section.* |
| ☐ | ☒ | A description of all covariates tested |
| ☐ | ☒ | A description of any assumptions or corrections, such as tests of normality and adjustment for multiple comparisons |
| ☐ | ☒ | A full description of the statistical parameters including central tendency (e.g. means) or other basic estimates (e.g. regression coefficient) AND variation (e.g. standard deviation) or associated estimates of uncertainty (e.g. confidence intervals) |
| ☐ | ☒ | For null hypothesis testing, the test statistic (e.g. *F*, *t*, *r*) with confidence intervals, effect sizes, degrees of freedom and *P* value noted<br>*Give P values as exact values whenever suitable.* |
| ☐ | ☒ | For Bayesian analysis, information on the choice of priors and Markov chain Monte Carlo settings |
| ☐ | ☒ | For hierarchical and complex designs, identification of the appropriate level for tests and full reporting of outcomes |
| ☐ | ☒ | Estimates of effect sizes (e.g. Cohen's *d*, Pearson's *r*), indicating how they were calculated |

*Our web collection on statistics for biologists contains articles on many of the points above.*

## Software and code

Policy information about availability of computer code

| | |
|---|---|
| Data collection | DEPOSED |
| Data analysis | We used open source programs for data pre-processing and analysis. Data analyses are conducted using R (version 4.0.2) and Python (3.7.10) scripts.<br>An in-house pipeline developed at the Institut Curie Bioinformatics Core Facility, following standard analysis in the field and available at https://github.com/bioinfo-pf-curie/RNA-seq was used for bulk RNA-seq data pre-prossessing.<br>DESeq2 (version 1.30.1) Bioconductor package was used for variance stabilization and differential analyses of bulk RNA-seq data.<br>The ComplexHeatmap (version 2.6.2) Bioconductor package was used for hierarchical clustering and heatmap visualization of gene expression level.<br>The ConsensusClusterPlus (version 1.54.0) Bioconductor package was used for consensus clustering analyses.<br>The umap CRAN packages was used for UMAP visualization.<br>The mixOmics framework (version 6.14.1) Bioconductor package was used to conduct Sparse Partial Least Squares Discriminant Analysis.<br>The ESTIMATE (version 1.0.11) R-Forge package to estimate Immune and stromal cells infiltration.<br>The immunedeconv (version 2.0.4) R Bioconductor package was used to compute the relative fraction of immune cells. The web application available in https://www.gsea-msigdb.org/gsea/msigdb/annotate.jsp, the GSEA tool (version 2.2.3) on the GO:BP gene set collection (version 7.4) and the GSVA (version 1.38.2) bioconductor package and the msigdbr (version 7.4.1) CRAN database package were used for functional enrichment analyses.<br>The affy (version 1.70.0) Bioconductor R package were used to normalize Affymetrix data.<br>The SVA (version 3.40.0) Bioconductor R package was used for cross-species and cross-plateform data integration.<br>The RnBeads package (version 1.6.1) Bioconductor package was used for DNA methylation array (Infinium methylationEPIC) data pre-processing and analysis. |

The mixKernel (version 0.7) CRAN package was used for Gene expression (bulk RNA-seq) and DNA methylation (EPIC array) data integration.
Seurat version 3 was used for single cell data pre-processing and analysis.
The pyscenic programm (version 0.10.3) was used to conduct Gene regulatory network analysis of single cell data.
ElPiGraph (version 1.0.0) and Monocle3 (version 0.2.3.0) R package were used to conduct trajectory inference analysis of single cell data.
The scVelo version 0.2.3 was used for RNA velocity analysis.
The cellphonedb binary (version 2.1.7) was run for ligand-receptor interaction analysis.
The CytoTRACE44 tool (version 0.3.3) was used to estimate cell differentiation state in single cell data.
The nebulosa R package (Version 1.0.1) was used to estimate the kernel gene-weighted density on single-nuclei data.

For manuscripts utilizing custom algorithms or software that are central to the research but not yet described in published literature, software must be made available to editors and reviewers. We strongly encourage code deposition in a community repository (e.g. GitHub). See the Nature Portfolio guidelines for submitting code & software for further information.

## Data

Policy information about availability of data

All manuscripts must include a data availability statement. This statement should provide the following information, where applicable:

- Accession codes, unique identifiers, or web links for publicly available datasets
- A description of any restrictions on data availability
- For clinical datasets or third party data, please ensure that the statement adheres to our policy

Raw and processed data included in this study have been deposited in NCBI's Gene Expression Omnibus and accessible through GEO SuperSeries accession number GSE242090 [https://www.ncbi.nlm.nih.gov/geo/query/acc.cgi?acc=GSE242090].
Cell lines bulk RNA-seq data are available through GEO accession number GSE241733 [https://www.ncbi.nlm.nih.gov/geo/query/acc.cgi?acc=GSE241733].
Bulk RNA-seq data of mouse rhabdoid tumor model are accessible through GEO accession number GSE241734 [https://www.ncbi.nlm.nih.gov/geo/query/acc.cgi?acc=GSE241734].
Mouse gene expression array (affimetrix) from Han et al., 2016 are available in GEO under accession number GSE64019.
Single cell RNA-seq data from mouse model are deposited in GEO under accession code GSE241736 [https://www.ncbi.nlm.nih.gov/geo/query/acc.cgi?acc=GSE241736]
Single cell RNA-seq data from human primary ATRT are deposited in GEO under accession code GSE241737 [https://www.ncbi.nlm.nih.gov/geo/query/acc.cgi?acc=GSE241737]
Bulk RNA-seq data of human primary ATRT are accessible through GSE241831 [https://www.ncbi.nlm.nih.gov/geo/query/acc.cgi?acc= GSE241736] accession code.
Bulk RNA-seq data from Andrianteranagna et al., 2021 are stored in GEO under accession code GSE175891 [https://www.ncbi.nlm.nih.gov/geo/query/acc.cgi?acc=GSE175891]. Bulk RNA-seq data from Leruste et al., 2019 are deposited in dbGaP database under accession code phs001915.v1.p1.
DNA methylation array (Illumina Infinium MethylationEPIC) data are accessible through GSE242089 [https://www.ncbi.nlm.nih.gov/geo/query/acc.cgi?acc=GSE242089] code. DNA methylation array data from Andrianteranagna et al, 2021 are available in GEO under accession number GSE175892 [https://www.ncbi.nlm.nih.gov/geo/query/acc.cgi?acc= GSE175892].
Source data are provided with the manuscript, without restriction.

## Research involving human participants, their data, or biological material

Policy information about studies with human participants or human data. See also policy information about sex, gender (identity/presentation), and sexual orientation and race, ethnicity and racism.

| | |
|---|---|
| Reporting on sex and gender | Sex information for mice are reported in the Supplementary Table 3. No sex nor gender information is reported for patients since it is not useful for the study. |
| Reporting on race, ethnicity, or other socially relevant groupings | none |
| Population characteristics | Children less than 18 years old, ATRT |
| Recruitment | Local retrospective series based on the histopathologlogical diagnosis made in our network; prospective analysis of fresh tumors with the appropriate diagnosis. We analyse all tumours diagnosed in our network centrally without any selection. |
| Ethics oversight | Freshly resected and snap-frozen human ATRT samples were collected following written informed consent of parents regarding tumor banking and use for research; approval of these consents was obtained by the internal review board from Curie Institute and Necker Hospital for Sick Children (Paris, France, IRB approved protocol number DC-2009-955). |

Note that full information on the approval of the study protocol must also be provided in the manuscript.

# Field-specific reporting

Please select the one below that is the best fit for your research. If you are not sure, read the appropriate sections before making your selection.

☒ Life sciences ☐ Behavioural & social sciences ☐ Ecological, evolutionary & environmental sciences

For a reference copy of the document with all sections, see nature.com/documents/nr-reporting-summary-flat.pdf

# Life sciences study design

All studies must disclose on these points even when the disclosure is negative.

| | |
|---|---|
| Sample size | The number of included tumors was limited by the rarity of the tumor but sufficient to draw conclusion as demonstrated by the statistical robustness of the results. Sample size: 49 human bulk RNA-seq samples, 54 human DNA methylation array samples, 16 mouse bulk RNA-seq sample, 3 human single cell RNA-seq samples, 3 mouse single cell RNA-seq samples, 12 cell line bulk RNA-seq samples were included in the study. |
| Data exclusions | no data was excluded, they pass all quality control for DNA methylation and RNAseq as assessed by the plateforms generating the data.. |
| Replication | no replication for the retrospective tumor omic data, experiment replication have been performed on cell line and mouse samples, human single cell data analysis results are confirmed with external public data. |
| Randomization | The study does not applied statistical analyses in which randomization is required. |
| Blinding | The study does not applied statistical analyses in which blinding is required. |

# Behavioural & social sciences study design

All studies must disclose on these points even when the disclosure is negative.

| | |
|---|---|
| Study description | *Briefly describe the study type including whether data are quantitative, qualitative, or mixed-methods (e.g. qualitative cross-sectional, quantitative experimental, mixed-methods case study).* |
| Research sample | *State the research sample (e.g. Harvard university undergraduates, villagers in rural India) and provide relevant demographic information (e.g. age, sex) and indicate whether the sample is representative. Provide a rationale for the study sample chosen. For studies involving existing datasets, please describe the dataset and source.* |
| Sampling strategy | *Describe the sampling procedure (e.g. random, snowball, stratified, convenience). Describe the statistical methods that were used to predetermine sample size OR if no sample-size calculation was performed, describe how sample sizes were chosen and provide a rationale for why these sample sizes are sufficient. For qualitative data, please indicate whether data saturation was considered, and what criteria were used to decide that no further sampling was needed.* |
| Data collection | *Provide details about the data collection procedure, including the instruments or devices used to record the data (e.g. pen and paper, computer, eye tracker, video or audio equipment) whether anyone was present besides the participant(s) and the researcher, and whether the researcher was blind to experimental condition and/or the study hypothesis during data collection.* |
| Timing | *Indicate the start and stop dates of data collection. If there is a gap between collection periods, state the dates for each sample cohort.* |
| Data exclusions | *If no data were excluded from the analyses, state so OR if data were excluded, provide the exact number of exclusions and the rationale behind them, indicating whether exclusion criteria were pre-established.* |
| Non-participation | *State how many participants dropped out/declined participation and the reason(s) given OR provide response rate OR state that no participants dropped out/declined participation.* |
| Randomization | *If participants were not allocated into experimental groups, state so OR describe how participants were allocated to groups, and if allocation was not random, describe how covariates were controlled.* |

# Ecological, evolutionary & environmental sciences study design

All studies must disclose on these points even when the disclosure is negative.

| | |
|---|---|
| Study description | *Briefly describe the study. For quantitative data include treatment factors and interactions, design structure (e.g. factorial, nested, hierarchical), nature and number of experimental units and replicates.* |
| Research sample | *Describe the research sample (e.g. a group of tagged Passer domesticus, all Stenocereus thurberi within Organ Pipe Cactus National Monument), and provide a rationale for the sample choice. When relevant, describe the organism taxa, source, sex, age range and any manipulations. State what population the sample is meant to represent when applicable. For studies involving existing datasets, describe the data and its source.* |
| Sampling strategy | *Note the sampling procedure. Describe the statistical methods that were used to predetermine sample size OR if no sample-size calculation was performed, describe how sample sizes were chosen and provide a rationale for why these sample sizes are sufficient.* |
| Data collection | *Describe the data collection procedure, including who recorded the data and how.* |
| Timing and spatial scale | *Indicate the start and stop dates of data collection, noting the frequency and periodicity of sampling and providing a rationale for* |

| | |
|---|---|
| Timing and spatial scale | *these choices. If there is a gap between collection periods, state the dates for each sample cohort. Specify the spatial scale from which the data are taken* |
| Data exclusions | *If no data were excluded from the analyses, state so OR if data were excluded, describe the exclusions and the rationale behind them, indicating whether exclusion criteria were pre-established.* |
| Reproducibility | *Describe the measures taken to verify the reproducibility of experimental findings. For each experiment, note whether any attempts to repeat the experiment failed OR state that all attempts to repeat the experiment were successful.* |
| Randomization | *Describe how samples/organisms/participants were allocated into groups. If allocation was not random, describe how covariates were controlled. If this is not relevant to your study, explain why.* |
| Blinding | *Describe the extent of blinding used during data acquisition and analysis. If blinding was not possible, describe why OR explain why blinding was not relevant to your study.* |

Did the study involve field work? ☐ Yes ☒ No

## Field work, collection and transport

| | |
|---|---|
| Field conditions | *Describe the study conditions for field work, providing relevant parameters (e.g. temperature, rainfall).* |
| Location | *State the location of the sampling or experiment, providing relevant parameters (e.g. latitude and longitude, elevation, water depth).* |
| Access & import/export | *Describe the efforts you have made to access habitats and to collect and import/export your samples in a responsible manner and in compliance with local, national and international laws, noting any permits that were obtained (give the name of the issuing authority, the date of issue, and any identifying information).* |
| Disturbance | *Describe any disturbance caused by the study and how it was minimized.* |

# Reporting for specific materials, systems and methods

We require information from authors about some types of materials, experimental systems and methods used in many studies. Here, indicate whether each material, system or method listed is relevant to your study. If you are not sure if a list item applies to your research, read the appropriate section before selecting a response.

### Materials & experimental systems

| n/a | Involved in the study | |
|---|---|---|
| ☐ | ☒ | Antibodies |
| ☐ | ☒ | Eukaryotic cell lines |
| ☒ | ☐ | Palaeontology and archaeology |
| ☐ | ☒ | Animals and other organisms |
| ☐ | ☒ | Clinical data |
| ☒ | ☐ | Dual use research of concern |
| ☒ | ☐ | Plants |

### Methods

| n/a | Involved in the study | |
|---|---|---|
| ☒ | ☐ | ChIP-seq |
| ☒ | ☐ | Flow cytometry |
| ☒ | ☐ | MRI-based neuroimaging |

## Antibodies

| | |
|---|---|
| Antibodies used | programmed death-ligand 1 (PD-L1) (1:100, clone E1L3N, Cell Signaling Technology, Beverly, USA), PD-1 (1:250, clone EPR4877(2), Epitomics, Cambridge, USA), CD3 (1:50, clone F7.2.38, Dako, Carpinteria, USA), CD4 (1:80, clone 4B12, Leica Biosystems, Wetzlar, Germany), CD8 (1:25, clone C8/144B, Thermo Scientific, Waltham, USA), CD45 (1:500, clone PD7/26 and 2B11, Dako, Carpinteria, USA), CD57 (1:40, clone HNK-1, BD Biosciences, Franklin Lakes, USA), CD68 (1:400, clone KP1, Dako, Carpinteria, USA), CD163 (1:50, clone IHC163, Diagomics, Blagnac, France), FOXP3 (1:50, clone 206D, BioLegend, USA), Granzyme B (ready to use, clone 11F1, Leica Biosys-tems, Wetzlar, Germany), OX40 (1:100, clone ACT-35, Thermo Scientific, Waltham, USA), OTX2 (1:600, clone 1H12C4B5, Thermo Fisher, Rockford, USA) and DCX (1:200, clone EPR19997, Abcam, Cambridge, United King-dom). monoclonal rabbit cleaved Notch1 (#4147, Cell signaling) and HRP-conjugated GAPDH (#HRP-60004, proteintech) antibodies. anti-rabbit immunoglobulin G horseradish peroxidase-coupled secondary antibody (1:3,000, NA934; Amersham Bio-sciences). |
| Validation | all antibodies used have been previously validated and published and are commercially available |

## Eukaryotic cell lines

Policy information about cell lines and Sex and Gender in Research

| | |
|---|---|
| Cell line source(s) | ATRT cell line CHLA-02-ATRT (#CRL-3020, ATCC) , IC-032 cell line, established in Curie Institute from a supra-tentorial ATRT |
| Authentication | no specific authentification was done |
| Mycoplasma contamination | negative |
| Commonly misidentified lines (See ICLAC register) | No commonly misidentified cell lines were used in the study |

## Animals and other research organisms

Policy information about studies involving animals; ARRIVE guidelines recommended for reporting animal research, and Sex and Gender in Research

| | |
|---|---|
| Laboratory animals | Mouse strain Smarcb1fl/fl R26 Cre (Han et al, 2016) |
| Wild animals | No |
| Reporting on sex | males or females |
| Field-collected samples | no field collected samples were used in the study |
| Ethics oversight | Approval for this study was received from Ministere de l'Enseignement Superieur et de la Recherche (authorization number 6,150). |

Note that full information on the approval of the study protocol must also be provided in the manuscript.

## Clinical data

Policy information about clinical studies

All manuscripts should comply with the ICMJE guidelines for publication of clinical research and a completed CONSORT checklist must be included with all submissions.

| | |
|---|---|
| Clinical trial registration | NCT04987476 |
| Study protocol | INNOVRT1 and INNOVRT 2; https://ichgcp.net/clinical-trial-registry/ NCT04987476 |
| Data collection | 2004 to 2019, Hopital Necker Enfants Malades |
| Outcomes | There are no outcomes in our work |
